# Metabolic response of *Brevibacterium epidermidis* TRM83610 to NaCl stress

**DOI:** 10.3389/fmicb.2026.1754185

**Published:** 2026-02-06

**Authors:** Tangliang Luo, Yafang Zhao, Lijun Wang, Zhanfeng Xia

**Affiliations:** 1 College of Life Science and Technology, Tarim University, Alar, Xinjiang, China; 2 Analysis and Testing Center, Tarim University, Alar, Xinjiang, China

**Keywords:** *Brevibacterium epidermidis*, ectoine, metabolomics, NaCl, response

## Abstract

To elucidate the metabolic response of *Brevibacterium epidermidis* TRM83610 to NaCl stress and promote its industrial application, this study employed metabolomics techniques to analyze changes in intracellular metabolites—particularly compatible solutes—under NaCl concentrations of 0, 5, 10, and 15%. Response surface methodology was further applied to optimize key fermentation parameters including carbon and nitrogen source concentrations, composite salt concentration, pH, and temperature, in order to evaluate the strain’s ectoine production capacity. The results revealed significant metabolic differences among the salinity treatment groups, with various secondary metabolites associated with antimicrobial activity and plant growth promotion being detected. Six compatible solutes dominated by ectoine were identified, among which Nε-acetyl-L-lysine was reported for the first time in the genus *Brevibacterium*. The metabolic strategies adopted by the strain in response to NaCl stress included osmoadaptation, oxidative stress resistance, and competition for survival. Through response surface optimization, the ectoine titer reached 440.60 mg/L, representing a 6.22-fold increase over the initial yield of 70.75 mg/L and demonstrating considerable application potential. This study enriches the metabolic profile of *B. epidermidis* TRM83610, preliminarily reveals its metabolic adaptation mechanisms under NaCl stress, and provides a theoretical basis for its further development and utilization.

## 1 Introduction

Under the backdrop of the Fourth Industrial Revolution, traditional chemical engineering-based manufacturing increasingly conflicts with sustainable development principles. In contrast, next-generation industrial biotechnology (NGIB) offers an eco-friendly solution to reduce production costs, conserve energy, mitigate emissions, and enable efficient biosynthesis (Ma et al., 2020; Chen and Tan, 2024). NGIB employs extremophilic microorganisms as chassis cells for biomanufacturing, with halophilic/halotolerant microbes gaining significant attention in recent years for green production strategies (Chen and Tan, 2024).

To date, researchers have developed multiple chassis cells from the genus *Halomonas* for novel biomanufacturing. *Halomonas bluephagenesis* TD01, a promising microbial cell factory, completed pilot-scale trials in 2024 for polyhydroxyalkanoate (PHA) production, demonstrating immense potential for low-cost, large-scale PHA synthesis (Zhang et al., 2025). Ma et al. (2020) enhanced ectoine titer to 28 g/L in *H. bluephagenesis* TD-ADEL-58 by integrating chromosomal *ectABC*, *lysC*, and asd genes, knocking out degradation-related genes, and implementing dynamic flux regulation via the LuxR-AHL and T7-like orthogonal systems. While the development of halophilic/halotolerant chassis cells underpins NGIB advancement (Wang et al., 2024), studies on ectoine biosynthesis remain predominantly focused on *Halomonas*, with limited exploration of other genera.

*Brevibacterium epidermidis* TRM83610, a halotolerant strain isolated from Mangya Emerald Lake on the Qinghai-Tibet Plateau, exhibits stable cellular morphology at 0–15% NaCl (Luo et al., 2025), robust growth at 30–40°C (Collins et al., 1983). This adaptability to environmental fluctuations may have driven the evolution of unique metabolic mechanisms, enabling the strain to produce industrially valuable metabolites, demonstrate a robust capacity for compatible solute accumulation (Echeveste Medrano et al., 2024; Vijaranakul et al., 1995), and potentially synthesize diverse compatible solutes (Abosamaha et al., 2022; Xing et al., 2024; Orhan et al., 2024). *B. epidermidis* has been proposed for amidase production and environmental bioremediation (Ruan et al., 2016a; Ruan et al., 2016b; Jin et al., 2008; Ziganshina et al., 2018; Esikova et al., 2023), highlighting its industrial relevance. However, its metabolic profile—particularly its metabolite responses to NaCl stress and associated regulatory networks—remains poorly characterized. This study aimed to elucidate the response of intracellular metabolites, particularly compatible solutes, in *B. epidermidis* under salt stress through metabolomic analysis. It also sought to supplement the metabolomic dataset for this industrially relevant strain and demonstrate its potential for ectoine synthesis.

In this study, we employed metabolomics to analyze intracellular metabolite levels in *B. epidermidis* TRM83610, under varying NaCl concentrations, with particular focus on abundance changes of compatible solutes supplemented by targeted metabolomic analysis. Additionally, fermentation conditions were optimized using response surface methodology to enhance ectoine production titer. This approach facilitates the elucidation of the strain’s metabolic regulatory network, thereby advancing understanding of its metabolic mechanisms for salt stress tolerance and promoting industrial applications. The research aims to reveal the metabolic response of *B. epidermidis* TRM83610 to NaCl stress while evaluating its ectoine production capacity, thereby establishing a foundational basis for future metabolic engineering efforts to develop this strain into a chassis cell for industrial utilization.

## 2 Materials and methods

### 2.1 Strain and cultivation

*B. epidermidis* TRM83610, isolated from Mangya Jade Lake on the Qinghai-Tibet Plateau, has been deposited in the China Center for Type Culture Collection (CCTCC) under the accession number CCTCC NO: M 20242137.

Medium: 10 g yeast extract, 7.5 g acid-hydrolyzed casein peptone, NaCl (as required), 1 L distilled water.

### 2.2 Untargeted metabolomics

Fermentation media were separately prepared with NaCl concentrations of 0, 5, 10, and 15%, each concentration comprising six biological replicates. Following sterilization via autoclaving at 1,211°C for 20 min and subsequent cooling, media were inoculated with 2% (v/v) seed culture. Cultivation proceeded for 6 days at 37°C with 150 rpm orbital shaking. Bacterial cells were subsequently harvested by centrifugation at 2,600 × *g* for 15 min, washed twice with isotonic NaCl solution via centrifugation, and the final pellet was stored overnight at −80°C. Lyophilized biomass was pulverized into homogeneous powder. Untreated *B. epidermidis* TRM 83610 (0 NaCl) served as the control group, while samples exposed to 5, 10, and 15% NaCl constituted treatment groups.

Untargeted metabolomic analysis was conducted by Shanghai Personal Biotechnology Co., Ltd. (Personalbio). Sample homogenization was performed by cryogenic grinding in liquid nitrogen. The samples were flash-frozen in liquid nitrogen for 5 min and then ground using a high-throughput tissue grinder (30 Hz, 60 s), repeated for 4 cycles. The resulting powder was subsequently blended uniformly using a 3D rotary mixer (25 rpm, 10 min). For each homogenized sample, the total homogenized powder was divided into aliquots of 50 mg each (otherwise, the entire powder was processed as a single aliquot). Each aliquot was transferred, at a solid-to-solvent ratio of 1:20 (w/v), into a 2 mL centrifuge tube containing 1 mL of pre-chilled 50% methanol. After vortex mixing for 30 s. The samples were then centrifuged at 4°C and 15,800 × *g* for 15 min. The supernatant from each tube (approximately 800 μL per tube) was collected, pooled, and concentrated to dryness under vacuum. The resulting residue was reconstituted in 150 μL of 50% methanol containing 5 ppm 2-chloro-L-phenylalanine (internal standard), vortexed for 30 s, and centrifuged again at 4°C and 15,800 × *g* for 10 min. All supernatant was collected, pooled, passed through a 0.22 μm filter, and transferred into an injection vial for subsequent analysis. Quality control (QC) samples were prepared by pooling 10–20 μL aliquots from each sample to monitor instrumental stability and data reliability.

Chromatographic separation employed an ACQUITY UPLC HSS T3 column (100Å, 1.8 μm, 2.1 × 100 mm) maintained at 40°C with 0.4 mL/min flow rate and 2 μL injection volume. Mobile phases consisted of (A) 0.1% formic acid in water and (B) acetonitrile containing 0.1% formic acid. The gradient program was: 0–1 min (5% A, 95% B); 1–7 min (5–95% A); 7–8 min (95% A, 5% B); 8.1–12 min (5% A, 95% B). High-resolution mass spectrometry operated in data-dependent acquisition (DDA) mode under Xcalibur software control (v4.7, Thermo Scientific) using a HESI ion source with spray voltage set at 3.5 kV. Key parameters included: sheath gas 40 arb, auxiliary gas 15 arb, capillary temperature 325°C, auxiliary gas heater 300°C. Full-scan MS1 spectra (m/z 100–1,000) were acquired at 60,000 resolution (AGC target standard, max IT 100 ms), with top-4 precursors selected for MS2 fragmentation at 15,000 resolution using 30% normalized collision energy, dynamic exclusion of 8 s, and automatic maximum injection time.

### 2.3 Targeted metabolomics

Qualitative detection of ectoine was performed by the Analysis and Testing Center of Tarim University. LC-MS conditions were as follows: An ACQUITY UPLC-BEH C18 column (1.7 μm, 2.1 × 100 mm) was employed for ultrahigh-performance liquid chromatography. Key parameters included capillary voltage 2.50 kV, source temperature 100°C, desolvation temperature 500°C, cone gas flow 50 L/h, desolvation gas flow 800 L/h, with mass scanning range set at m/z 50–2,000 and scan time 0.20 s.

Targeted metabolomic analysis was conducted by Yanxuan Biotechnology (Hangzhou) Co., Ltd. Samples were subsampled from those used in untargeted metabolomics analysis, with three biological replicates per group. LC-MS analysis based on selective multiple reaction monitoring (MRM) technology utilized a Shimadzu Nexera X2 LC-30AD UHPLC system. Mobile phases consisted of (A) 0.1% formic acid aqueous solution and (B) acetonitrile containing 0.1% formic acid. Chromatographic separation proceeded at 40°C column temperature with 300 μL/min flow rate and 1 μL injection volume. Mass spectrometric detection was performed on a 5,500 QTRAP mass spectrometer (AB Sciex) in positive ion mode. ESI source parameters were configured as follows: source temperature 550°C; ion source gas 1 (GS1): 55 psi; ion source gas 2 (GS2): 55 psi; curtain gas (CUR): 35 psi; ion spray voltage (IS): 5,500 V. Detection was carried out in MRM mode.

### 2.4 Experimental design

To determine the optimal combination and concentrations of carbon and nitrogen sources, the carbon sources evaluated included glucose, maltose, corn, oats, millet, soluble starch, and sucrose. The nitrogen sources tested were peptone, soy peptone, fish peptone, acid-hydrolyzed casein, beef extract, yeast extract, and ammonium sulfate. Since sodium glutamate is beneficial for ectoine synthesis, it was incorporated into the medium, and its optimal concentration was screened at levels of 0.02, 0.04, 0.06, 0.08, and 0.10 mol/L. The composite salt concentration was tested at 25, 50, 75, 100, 125, 150, and 175 g/L.

The fermentation conditions optimized included: fermentation duration (1–9 d), temperature (25, 28, 31, 34, 37, 40, and 43°C), shaking speed (100, 120, 140, 160, 180, and 200 r/min), initial pH of the fermentation medium (5.5, 6.5, 7.5, 8.5, 9.5, and 10.5), inoculation size (1–8%), and flask filling volume (50, 100, 150, 200, and 250 mL).

A Plackett-Burman experimental design with 12 runs was employed to screen five factors selected from the single-factor experiments, aiming to identify the most significant ones (complex salt concentration and fill volume). Subsequently, a steepest ascent experiment was conducted to approximate the optimal response region and determine the central point for further optimization. Finally, response surface methodology (RSM) was performed using Minitab 21 for optimization.

### 2.5 Data analysis

Raw data were preprocessed and subjected to quality control using the XCMS package in R, filtering out metabolites with RSD > 30%. Metabolite identification was performed by matching against public databases (HMDB, MassBank, LipidMaps, mzCloud, KEGG) and Biocode’s in-house metabolite library, with identification confidence levels set at Level 2 or above.

Relative abundance analysis of metabolites was conducted using ggplot2 (v3.4). Pairwise comparative differential analysis of sample data was performed using the Ropls R package. Multi-group comparative differential analysis employed the PMCMRplus (v1.9), Pheatmap (v1.0), and clusterProfiler (v4.6) packages in R. Multi-group differential volcano plots and association network diagrams were generated on the Biocode GeneCloud platform (accessed April 1, 2025).^1^

Standard curves, chromatograms of samples versus five compatible solute standards, DMs, and bar charts for fermentation optimization were plotted using Origin 2024 software. The metabolic network diagram was created with iodraw.^2^ Response surface plots and contour plots were generated using Minitab 21. Data are expressed as mean values.

## 3 Results

### 3.1 Non-targeted metabolomics analysis

#### 3.1.1 Quality control evaluation

**FIGURE 1:**
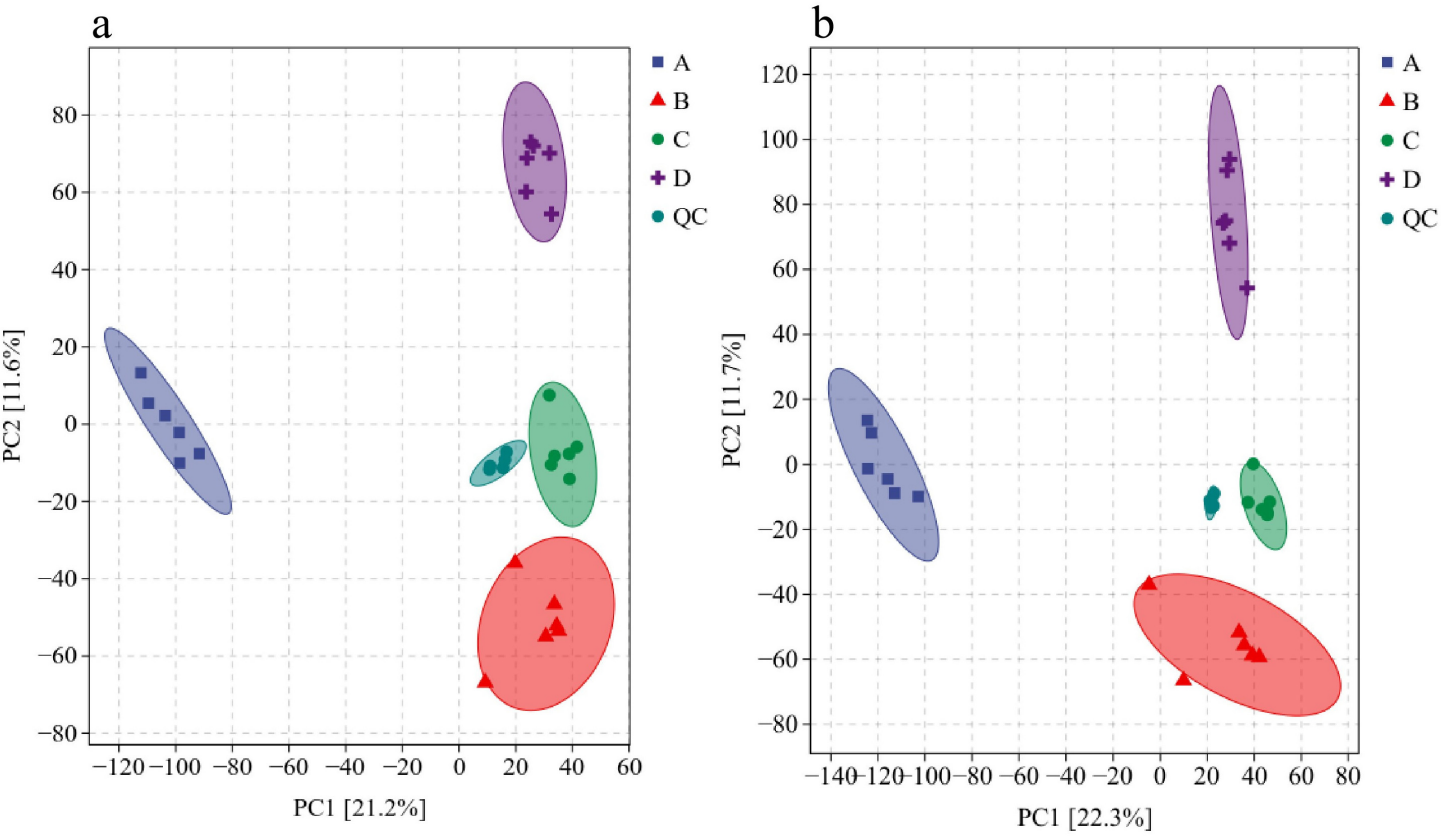
Principal component analysis (PCA) overview. **(a)** PCA in positive ion mode; **(b)** PCA in negative ion mode. Labels A–D denote NaCl concentrations of 0, 5, 10, and 15%, respectively.

As shown in Figure 1, the tight clustering of QC samples with minimal inter-sample variation confirmed the stability of the analytical methodology and instrumentation, ensuring reliable metabolite detection. Within-group samples for both control and treatment groups clustered within the 95% confidence interval, exhibiting low intra-group variability, while distinct inter-group separation underscored statistically significant metabolic differences between conditions.

#### 3.1.2 Impact of NaCl stress on intracellular metabolites in *B. epidermidis*

**FIGURE 2:**
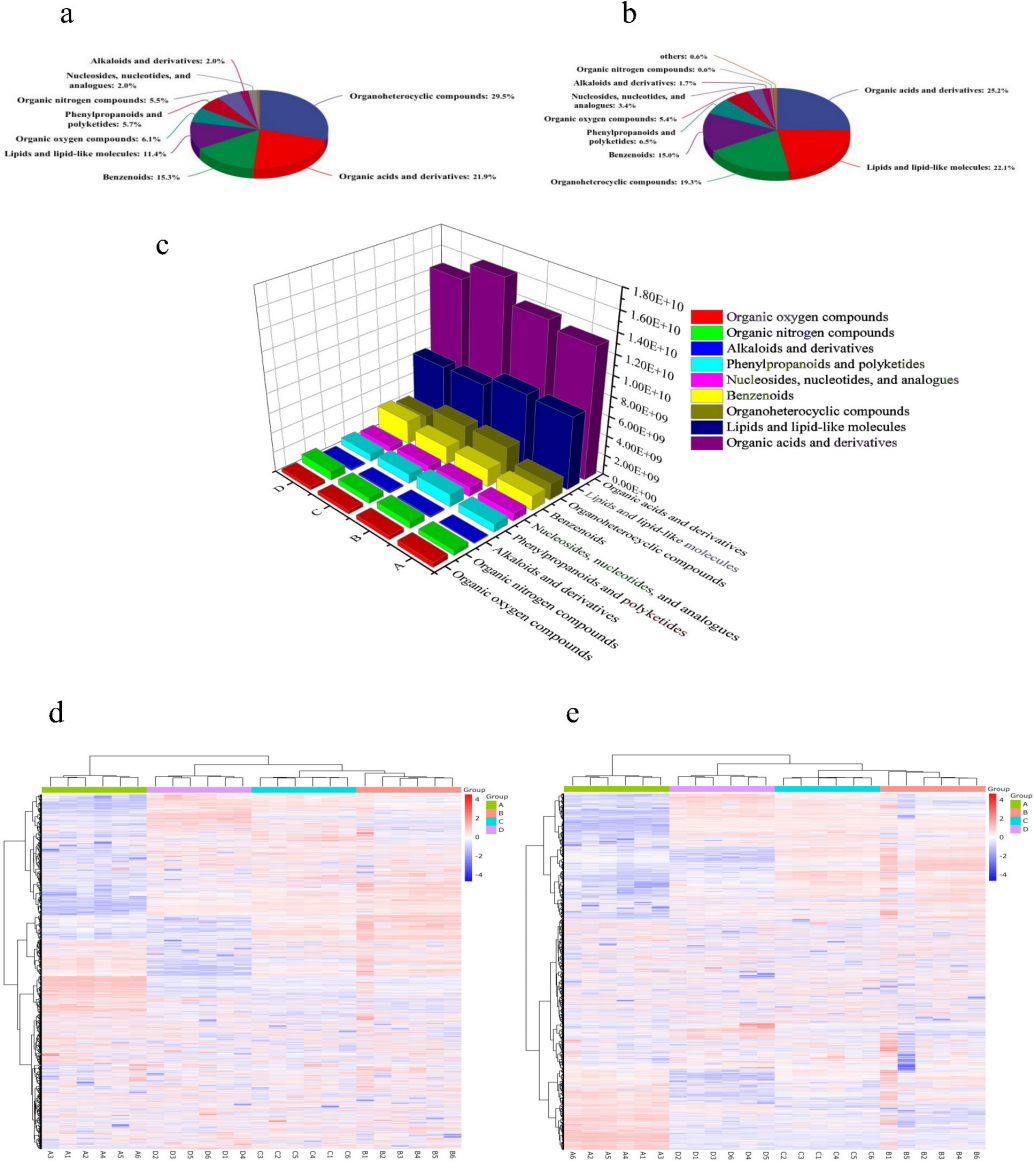
Differential metabolite distribution and hierarchical clustering analysis. **(a)** Superclass distribution of DMs in positive ion mode; **(b)** superclass distribution of DMs in negative ion mode; **(c)**: histogram of relative abundance changes for 9 compound classes; **(d)** hierarchical clustering in positive ion mode; **(e)** hierarchical clustering in negative ion mode. Labels A–D correspond to NaCl concentrations of 0, 5, 10, and 15%.

Metabolite identification via database matching using retention time, mass-to-charge ratio (m/z), and molecular weight revealed 1,416 metabolites, including 985 in positive ion mode and 431 in negative ion mode. Metabolites are primarily classified into nine major categories (Figures 2a,b). In positive ion mode (Figure 2a), the predominant metabolites were organic heterocyclic compounds (29.5%), organic acids and derivatives (21.9%), benzenoids (15.3%), lipids and lipid-like molecules (11.4%), oxygen-containing organic compounds (6.1%), phenylpropanoids and polyketides (5.7%), and nitrogen-containing organic compounds (5.5%). In negative ion mode (Figure 2b), the predominant metabolites were organic acids and derivatives (25.2%), lipids and lipid-like molecules (22.1%), organic heterocyclic compounds (19.3%), benzenoids (15.0%), phenylpropanoids and polyketides (6.5%), and oxygen-containing organic compounds (5.4%).

NaCl stress significantly altered the metabolic profile of *B. epidermidis* TRM83610. The total relative abundance of nine major metabolite classes was affected by NaCl stress (Figure 2c). When the NaCl concentration increased, the abundance of Organic oxygen compounds gradually decreased, while the abundance of benzenoids gradually increased. The abundances of the remaining classes all showed an initial increase followed by a decrease. Significant changes occurred in the relative abundance of intracellular metabolites across different NaCl concentrations (Figures 2d,e). Hierarchical clustering heatmap analysis revealed that the sample groups formed two distinct clusters: an initial branch without NaCl addition, and a second branch containing samples exposed to 5, 10, and 15% NaCl. Notably, the samples treated with 5 and 10% NaCl clustered closely together, indicating a high degree of similarity in their metabolic profiles, with significant but relatively small differences between them. This suggests that the metabolic response of the strain to 5 and 10% NaCl stress was similar, as evidenced by comparable numbers of differential metabolites (DMs). However, the relative abundances of metabolites still changed progressively with increasing NaCl concentration.

#### 3.1.3 Pairwise comparative differential analysis

Orthogonal partial least squares-discriminant analysis (OPLS-DA) permutation test plots (Supplementary Figure 1) demonstrated that all Q^2^ values for permuted models fell below the original Q^2^ value (far right), confirming the absence of overfitting and validating the model’s reliability. DMs analysis were subsequently performed using this robust model.

**FIGURE 3:**
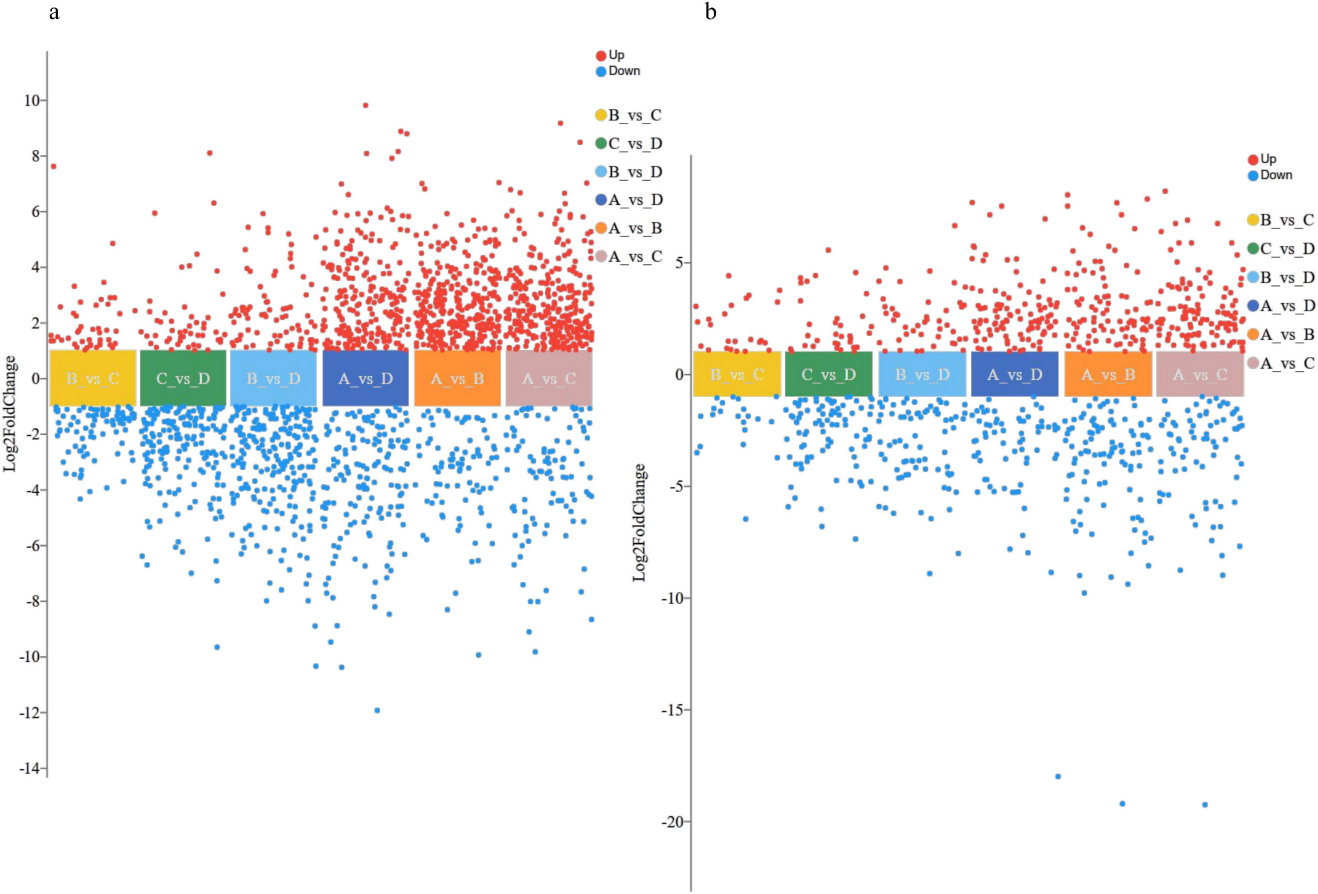
Multi-group differential volcano plots. Each dot represents a DM, with red indicating upregulation and blue downregulation. **(a)** Positive ion mode; **(b)** Negative ion mode. Labels A–D: NaCl concentrations of 0, 5, 10, and 15%. Comparisons (e.g., A vs. B) denote pairwise analysis between groups B and A.

DMs were filtered based on *P* < 0.05 and fold change > 2 or < 0.5, then visualized via multi-group differential volcano plots (Figure 3). Upregulated DMs progressively increased from left to right (Table 1). Total DMs initially rose and then declined with escalating NaCl concentrations, peaking at 10% NaCl (642 DMs) and 15% NaCl (632 DMs), with 434 and 422 DMs in positive ion mode and 208 and 210 in negative ion mode, respectively. The 5% vs. 10% NaCl comparison yielded the fewest DMs (189 total: 141 in positive, 48 in negative), indicating minimal metabolic divergence between these groups. Conversely, the control (0 NaCl) vs. 10% NaCl comparison exhibited the highest number of DMs, underscoring the strain’s strongest metabolic response under 10% NaCl stress.

**TABLE 1:** Number of DMs across sample groups.

Groups	B vs. C	C vs. D	B vs. D	A vs. D	A vs. B	A vs. C
Positive	141	247	322	422	383	434
Negative	48	125	133	210	156	208
Up	76	92	139	359	393	456
Down	113	280	316	273	146	186
Total	189	372	456	632	539	642

Numerical values indicate the count of differential metabolites.

**FIGURE 4:**
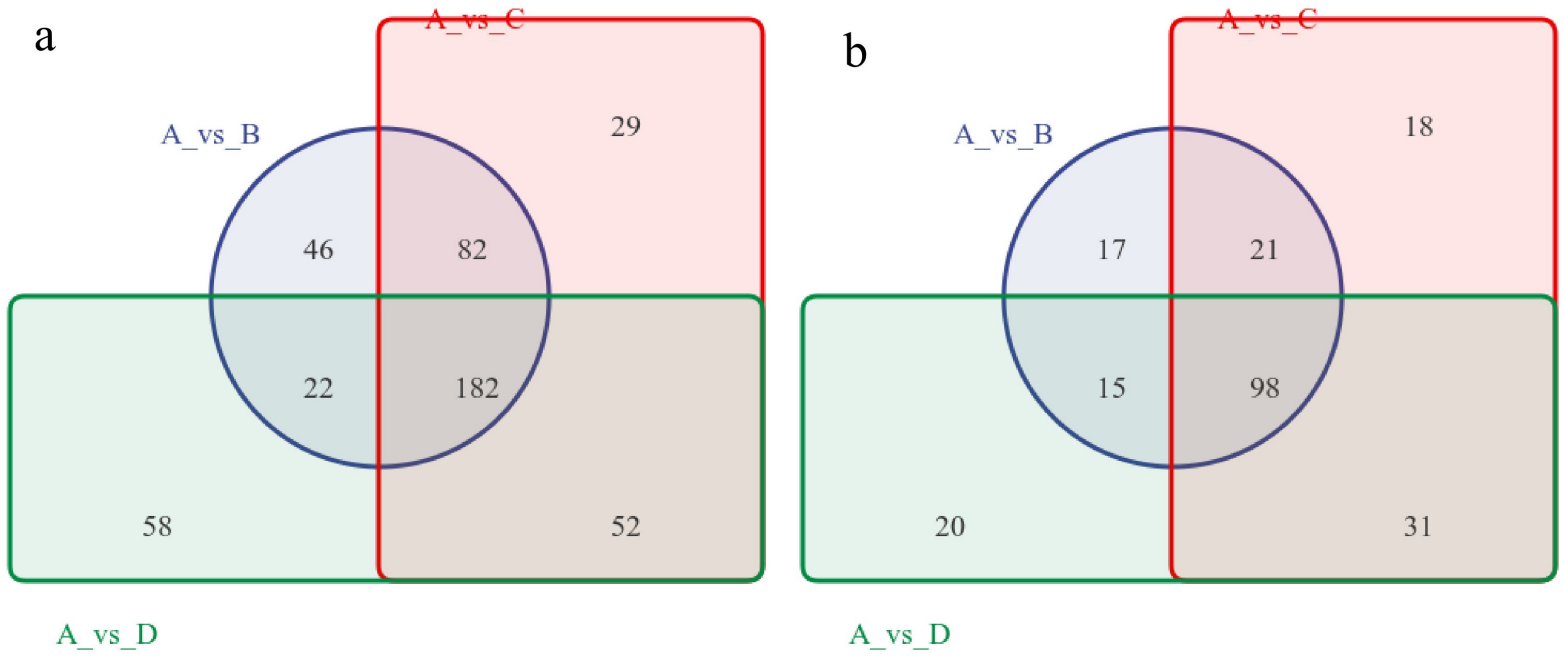
Gear Venn diagrams of differential metabolites. **(a)** Gear Venn diagram in positive ion mode; **(b)** Gear Venn diagram in negative ion mode. Labels A–D: NaCl concentrations of 0, 5, 10, and 15%. Comparisons (e.g., A vs. B) denote pairwise group analysis.

According to the criteria of *P* < 0.01, FDR < 0.05, fold change > 2 or < 0.5, and VIP > 1, differentially abundant metabolites (DMs) were screened. The Venn diagram of DMs (Figure 4) revealed that in positive ion mode, the treatment groups shared 182 DMs, with 82 DMs common between 5% NaCl and 10% NaCl, 22 DMs common between 5% NaCl and 15% NaCl, and 52 DMs common between 10% NaCl and 15% NaCl. In negative ion mode, the treatment groups shared 98 DMs, with 21 DMs common between 5% NaCl and 10% NaCl, 15 DMs common between 5% NaCl and 15% NaCl, and 31 DMs common between 10% NaCl and 15% NaCl. Analysis of unique DMs in each treatment group showed that in positive ion mode, 10% NaCl had the fewest unique DMs (29), while in negative ion mode, 5% NaCl had the fewest unique DMs (17), followed closely by 10% NaCl with 18 unique DMs.

#### 3.1.4 KEGG enrichment analysis

In the permutation test plot of the partial least squares-discriminant analysis (PLS-DA) (Supplementary Figure 2), all Q^2^ points in both positive and negative ion modes were lower than the original Q^2^ point on the far right, indicating that the model was reliable and effective without overfitting. Based on this model, multi-group comparative differential analysis of metabolites was conducted. Given the large number of differentially metabolites (DMs) identified in pairwise comparisons, these abundance changes might not entirely reflect NaCl concentration-responsive DMs, potentially interfering with the analysis process and experimental results and complicating the screening of critical metabolites. Therefore, in the multi-group comparative analysis, KEGG enrichment analysis was first applied to the differential metabolite sets to filter out most metabolites via thresholding.

**FIGURE 5:**
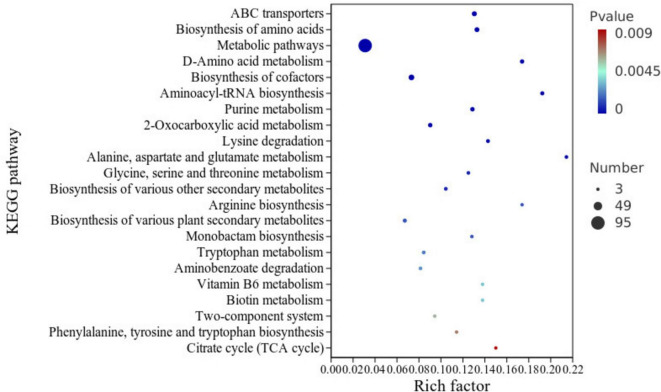
KEGG enrichment analysis bubble plot. *X*-axis: Enrichment ratio; *Y*-axis: Metabolic pathways. Color intensity reflects significance (blue: lower; red: higher). Dot size correlates with the number of enriched compounds.

By leveraging KEGG enrichment analysis, differentially abundant metabolites were mapped to specific metabolic pathways to identify key pathways and analyze their roles under NaCl stress. Subsequently, 22 metabolic pathways were screened from 96 candidate pathways using a threshold of *P* < 0.01 and visualized in a factor loading plot (Figure 5). As shown in Figure 5, the most significantly enriched pathway was ABC transporters, followed by Biosynthesis of amino acids. Enriched pathways related to amino acid metabolism included Lysine degradation, Alanine, aspartate and glutamate metabolism, Glycine, serine and threonine metabolism, Arginine biosynthesis, Phenylalanine, tyrosine and tryptophan biosynthesis, and D-Amino acid metabolism. These pathways regulate the synthesis or degradation of specific amino acids, meeting cellular demands for acidic substances under salt stress (Xing et al., 2024).

#### 3.1.5 Significantly enriched DMs and correlation analysis

A total of 102 DMs (Supplementary Table 1) were mapped to 22 significantly enriched metabolic pathways, encompassing compounds associated with osmoprotection, antioxidant activity, antimicrobial effects, anti-inflammatory properties, and plant growth promotion, highlighting the broad application potential of *B. epidermidis* TRM83610. The relative abundances of these compounds under different NaCl concentrations are presented in Supplementary Table 2.

**FIGURE 6:**
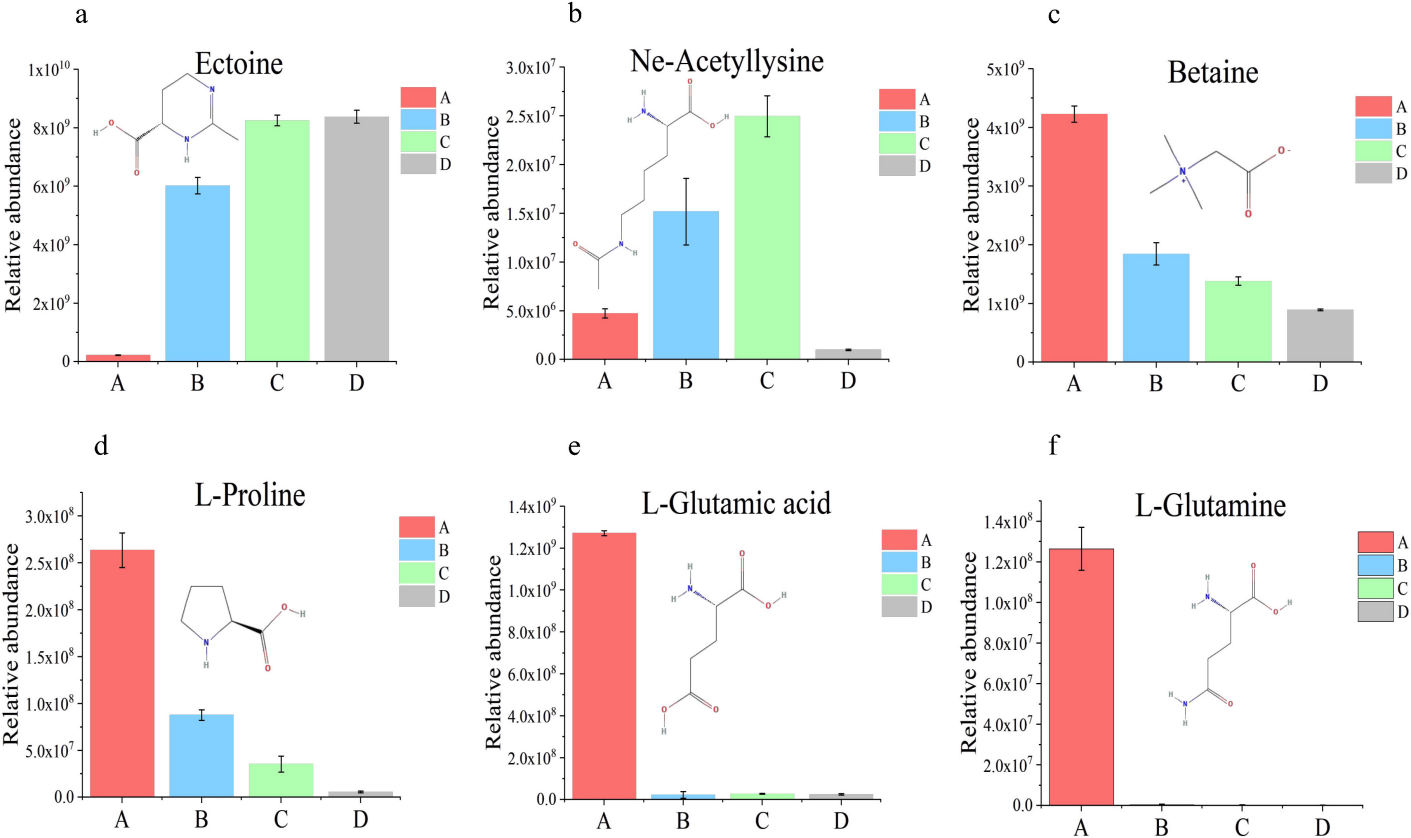
Potential compatible solutes and their abundance changes. **(a–f)** Bar charts represent the relative abundances of Ectoine, Nε-acetyl-L-lysine, L-glutamic acid, L-glutamine, L-proline, and betaine, respectively.

Compatible solutes are pivotal for halotolerant microorganisms to mitigate NaCl stress. Among the significantly enriched differentially metabolites (DMs), six potential compatible solutes were identified: ectoine, betaine, L-glutamic acid, L-glutamine, Nε-acetyl-L-lysine, and L-proline, with ectoine exhibiting a maximum relative abundance significantly higher than the others. These solutes have been validated to function as osmoprotectants in halotolerant or halophilic microbial cells (Schmidt and Bode, 1992; Saum et al., 2013), yet their relative abundance trends under varying NaCl concentrations were inconsistent (Figure 6). Specifically, as NaCl concentration increased, the relative abundance of ectoine progressively rose, peaking at 15% NaCl. In contrast, Nε-acetyl-L-lysine initially increased before declining, while betaine, L-glutamic acid, L-glutamine, and L-proline showed gradual reductions in relative abundance with elevated NaCl levels.

**FIGURE 7:**
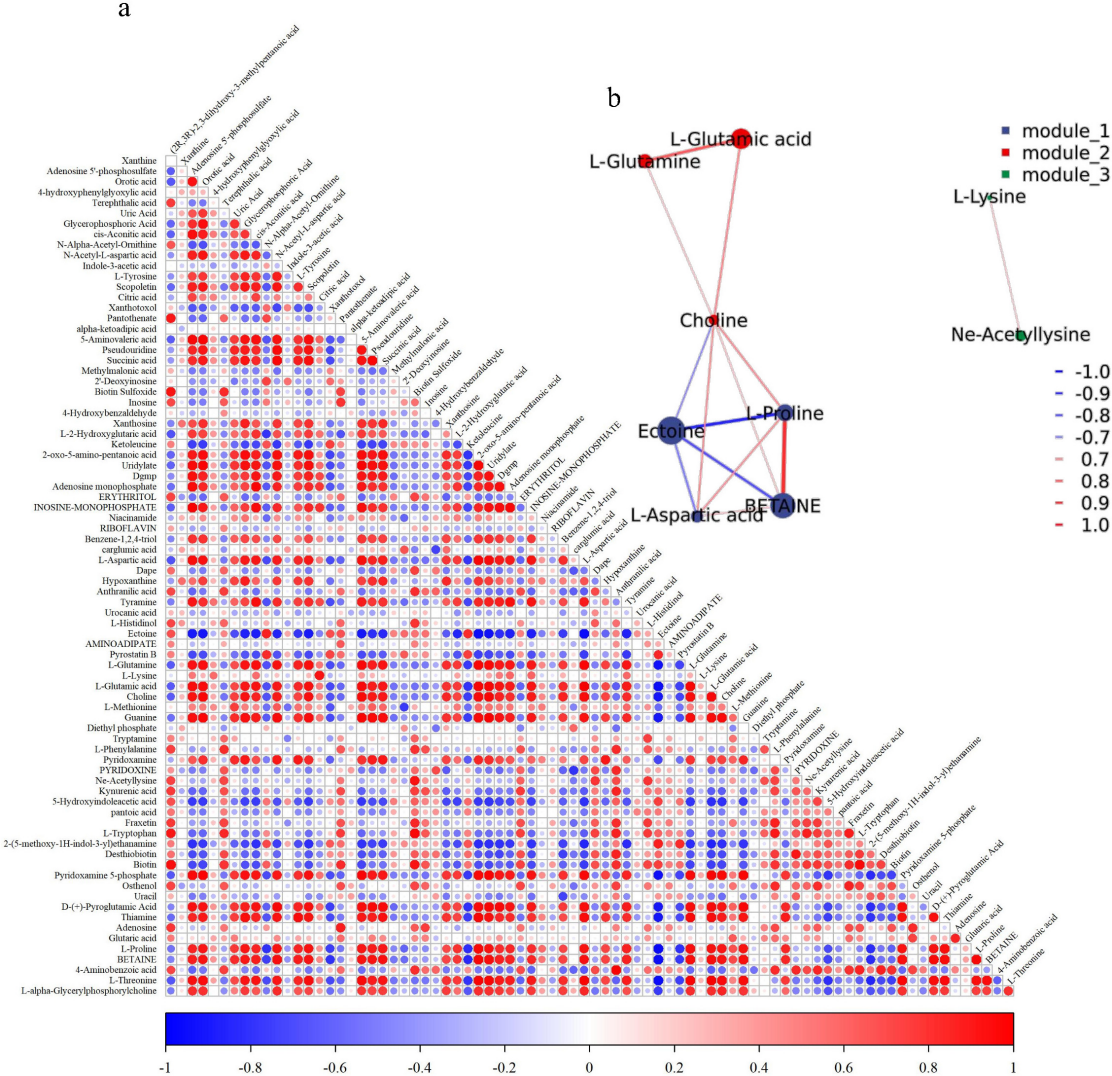
Correlation analysis of differentially abundant metabolites. Red indicates positive correlations, blue indicates negative correlations. **(a)** Global correlation analysis: darker colors denote stronger correlations. **(b)** Local correlation network analysis: thicker lines and darker colors represent stronger correlations; node size is proportional to the maximum relative abundance of compounds across different NaCl concentrations.

To analyze the correlations between significantly enriched differentially abundant metabolites (DMs) and ectoine, thereby revealing the major metabolite classes influencing ectoine synthesis and their associations with other compatible solutes, Spearman correlation coefficient analysis was performed on DMs (Supplementary Table 3) from the top 22 significantly enriched metabolic pathways. The results were visualized through a global correlation network (Figure 7a) and a local correlation network (Figure 7b).

Spearman correlation analysis revealed that 31 metabolites exhibited strong negative correlations with ectoine (*r*_*s*_ < −0.7), including 14 organic acids and derivatives, and 6 nucleosides, nucleotides, and analogs. Conversely, 6 metabolites showed strong positive correlations with ectoine (*r*_*s*_ > 0.7), among which 3 were indole and its derivatives. As illustrated in Figure 7a, ectoine and Nε-acetyl-L-lysine displayed negative correlations with betaine, L-proline, L-glutamic acid, L-glutamine, and L-aspartic acid. In contrast, betaine, L-proline, L-glutamic acid, L-glutamine, and L-aspartic acid were positively correlated with each other. Notably, Nε-acetyl-L-lysine showed no significant correlations with other compatible solutes.

#### 3.1.6 Metabolic network analysis

**FIGURE 8:**
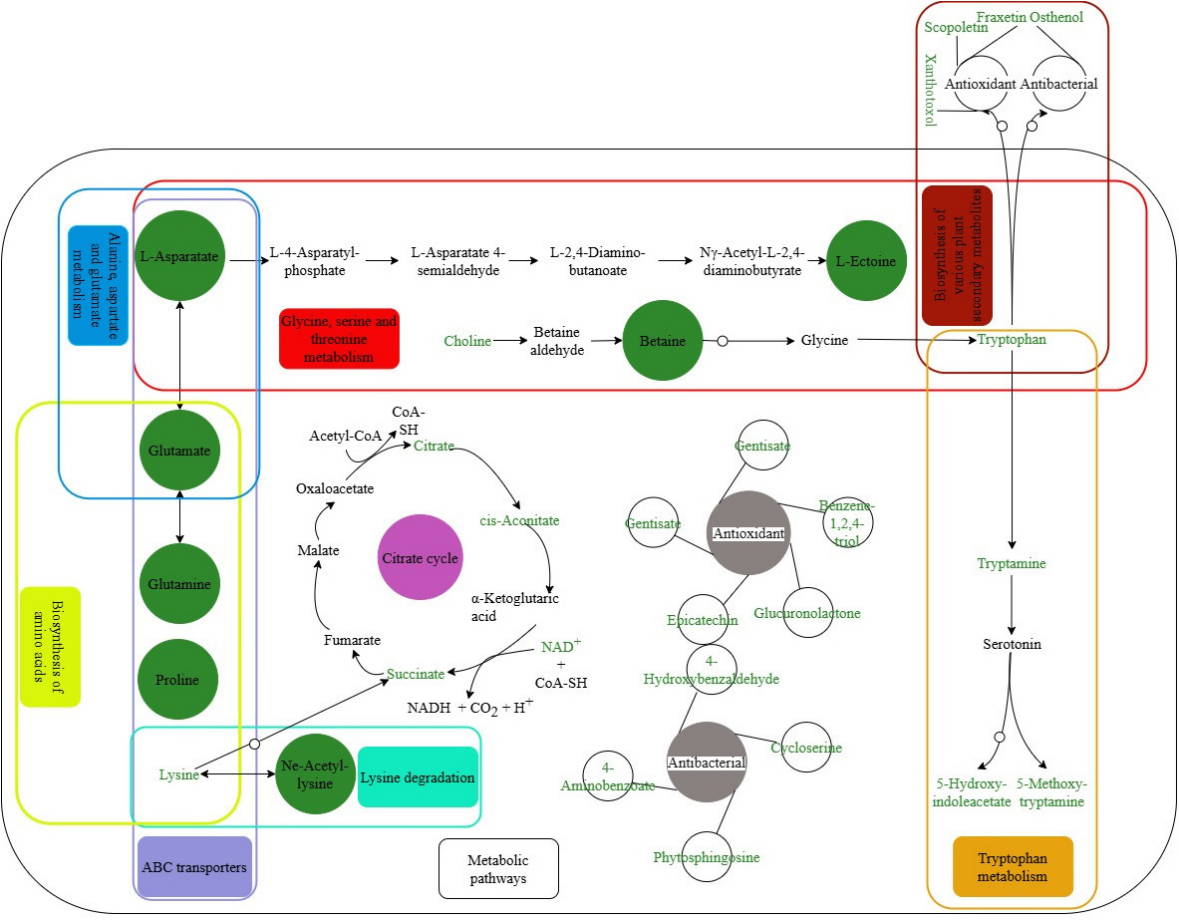
Metabolic network diagram between specific metabolites and KEGG Pathways. Green circles: represent specific metabolites; green text: detected metabolites; solid arrows: reactions defined in KEGG pathways; circular arrows: reactions involving intermediate products; rounded rectangles with distinct colors denote different metabolic pathways.

Based on the KEGG enrichment analysis results, a metabolic network diagram of specific metabolites was constructed using metabolite-pathway relationships from KEGG PATHWAY (Figure 8). The identified compatible solutes were tightlyinterconnected through multiple metabolic pathways. Cells uptake amino acids from the extracellular environment via ABC transporters. Aspartate serves as the substrate for ectoine synthesis, while glutamate provides amino groups for ectoine biosynthesis and can also be converted to aspartate as a supplementary source. Glutamine and proline are metabolized into glutamate to replenish its pool. Lysine metabolism generates Nε-acetyl-L-lysine, which undergoes deacetylation to regenerate lysine. Oxidative degradation of lysine ultimately produces succinate, which enters the tricarboxylic acid (TCA) cycle. Choline acts as the substrate for betaine synthesis, and betaine may be converted to glycine as a carbon source, subsequently forming tryptophan (Thomas et al., 2025), which is further metabolized into 5-hydroxyindoleacetate and 5-methoxytryptamine.

### 3.2 Targeted identification analysis

#### 3.2.1 Targeted identification of intracellular compatible solutes in *B. epidermidis*

**FIGURE 9:**
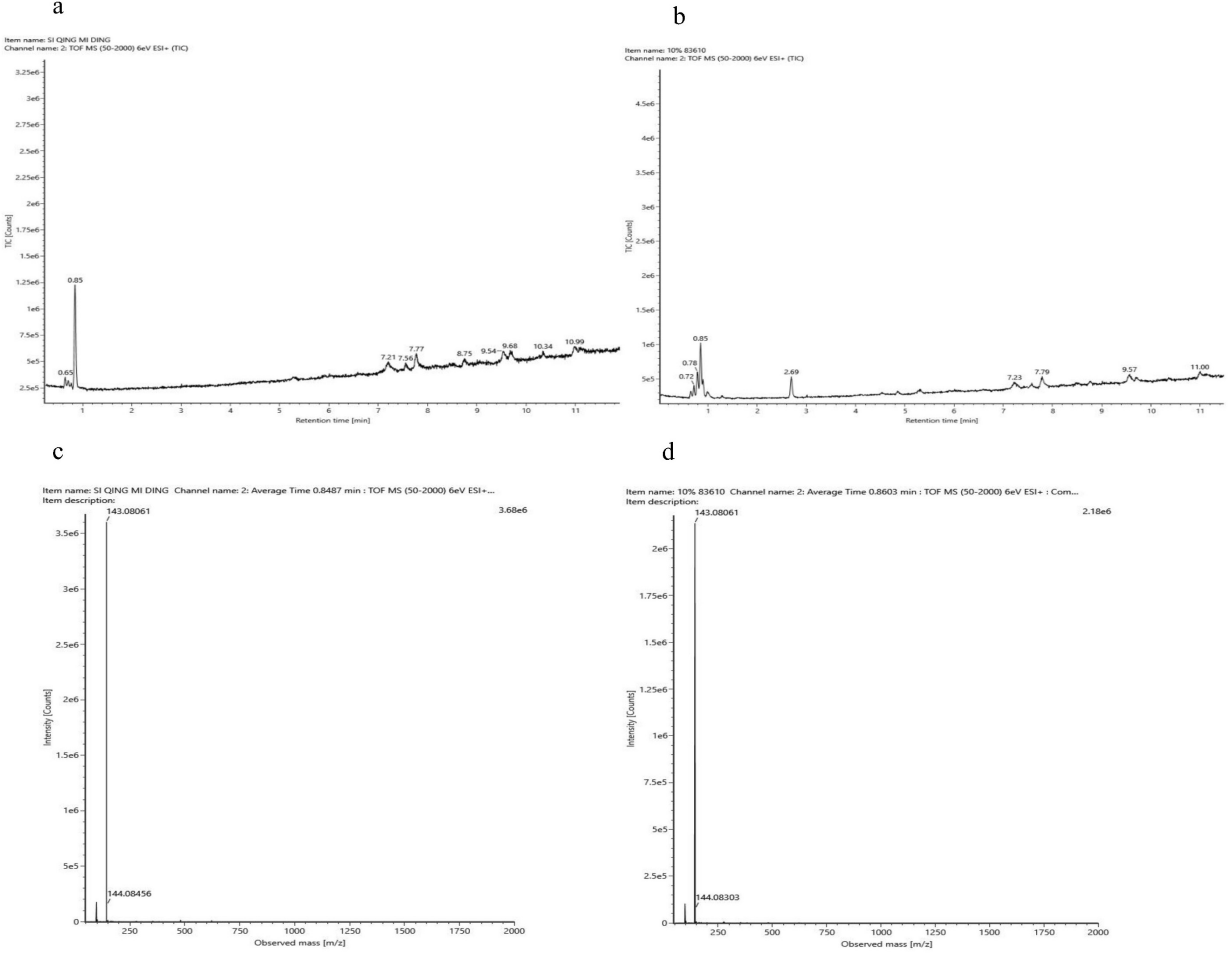
Total Ion chromatograms (TIC) and mass spectra of the sample and ectoine standard. **(a)** Total ion chromatogram of the ectoine standard; **(b)** total ion chromatogram of the sample; **(c)** mass spectrum of the ectoine standard; **(d)** mass spectrum of the sample.

Untargeted metabolomics analysis preliminarily indicated that the primary compatible solute in TRM83610 might be ectoine. To confirm this identification, LC-MS was employed to analyze the intracellular extract of TRM83610. Comparative assessment of retention times and mass-to-charge ratios (m/z) with an ectoine standard (Figure 9) demonstrated that TRM83610 produces ectoine.

#### 3.2.2 Quality control assessment in targeted metabolomics

**FIGURE 10:**
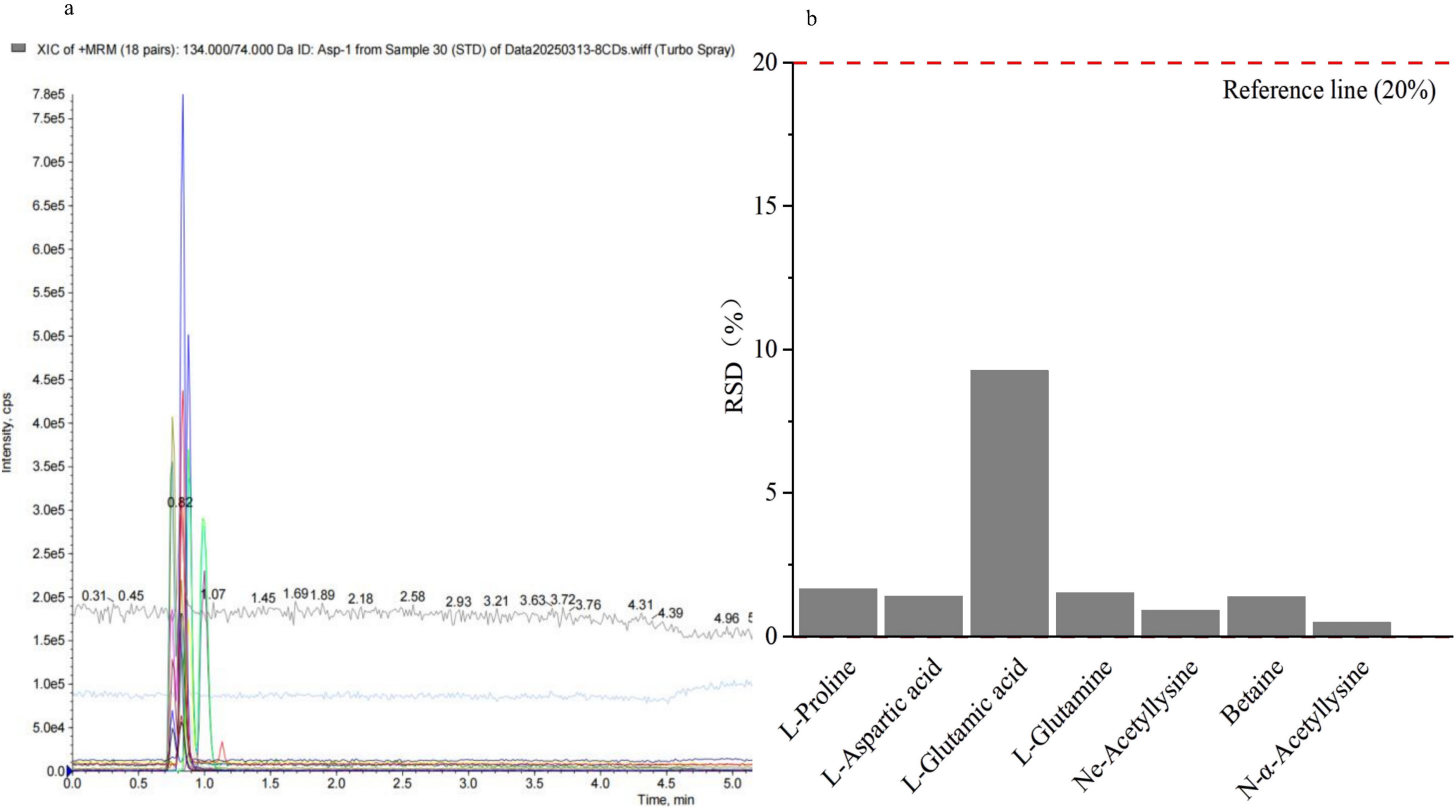
Extracted ion chromatograms and RSD plot of samples. **(a)**: Extracted ion chromatogram (EIC) of the standards; **(b)**: Relative standard deviation (RSD) of quality control (QC) samples.

The extracted ion chromatograms (EIC) of the standards (Figure 10a) demonstrate satisfactory chromatographic separation, with sharp and symmetrical peaks, enabling mass spectrometric quantitative analysis of the metabolites. The relative standard deviation (RSD) of the quality control (QC) samples was < 20% (Figure 10b), indicating stable and reliable sample data. These experimental samples are suitable for qualitative and quantitative detection analyses of several compatible solutes.

#### 3.2.3 Targeted metabolomics analysis

**FIGURE 11:**
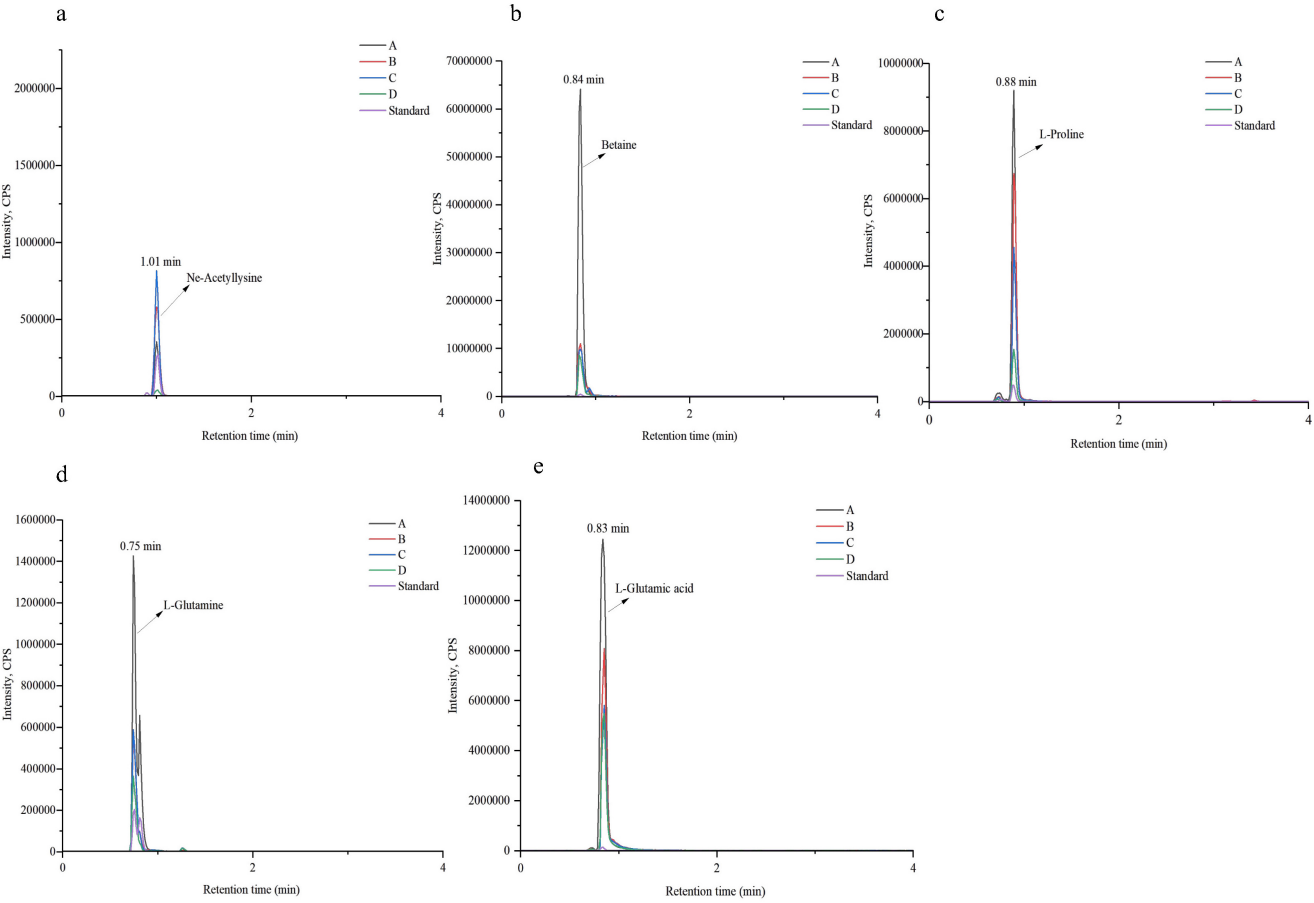
Chromatograms of the sample and five standards. **(a–e)** Chromatograms of the standard and sample for Nε-Acetyl-L-lysine, betaine, L-proline, L-glutamine, and L-glutamic acid, respectively.

LC-MS coupled with MRM mode was employed to detect five potential compatible solutes in the samples, including Nε-acetyl-L-lysine, and quantitative analysis of the detected compounds was performed based on standard curves of the respective reference standards (Supplementary Figure 3). As shown in Figure 11, five compounds in the sample exhibited product ion retention times identical to those of the Nε-acetyl-L-lysine, betaine, L-proline, L-glutamic acid, and L-glutamine standards, confirming the intracellular presence of these solutes in TRM 83610. Table 2 reveals that the levels of betaine, L-proline, L-glutamic acid, and L-glutamine gradually decreased with increasing NaCl concentrations. Notably, the Nε-acetyl-L-lysine content increased within the 0–10% NaCl range but dropped sharply at 15% NaCl.

**TABLE 2:** Content of compatible solutes in sample groups.

ID	Name	Content (mg/kg)				
		L-Proline	L-Glutamic acid	L-Glutamine	Nε−Acetyl-L-lysine	Betaine
1	A1	507.1	13,890	77.7	25.4	5,984
2	A2	506.8	14,399	72.9	28.1	5,628
3	A3	514.6	14,302	48.0	20.5	5,874
4	B1	246.9	2,248	12.2	27.4	1,097
5	B2	238.2	1,700	8.8	26.4	999
6	B3	281.7	1,810	10.7	33.5	986
7	C1	92.0	1,129	5.77	42.0	514
8	C2	147.3	1,491	5.46	46.6	643
9	C3	180.1	1,835	8.22	45.4	818
10	D1	36.9	999	2.86	0.63	389
11	D2	60.3	1,510	3.44	0.30	518
12	D3	57.1	1,403	3.68	0.40	505

(A–D): Represent NaCl concentrations of 0, 5, 10, and 15%, respectively.

### 3.3 Optimization of ectoine production in *B. epidermidis* using response surface methodology

#### 3.3.1 Plackett-Burman design results

A 12-run Plackett-Burman design was implemented using Minitab 21 software to evaluate eight factors listed in Table 3 (see Table 4 for experimental design details). Fermentation cultures were performed under the 12 distinct factor combinations specified by the design. Ectoine content in the bacterial biomass was subsequently quantified via high-performance liquid chromatography (HPLC).

**TABLE 3:** Factors and levels in the Plackett-Burman experimental design.

Factor no.	Factors	Low	High
X_1_	Sucrose	1 g/L	5 g/L
X_2_	Soybean peptone	20 g/L	30 g/L
X_3_	Sodium glutamate	6.76 g/L	13.52 g/L
X_4_	Complex salt concentration	100 g/L	150 g/L
X_5_	Medium fill volume	50 mL	150 mL
X_6_	Temperature	31°C	37°C
X_7_	Inoculation volume	4%	6%
X_8_	Fermentation time	4 d	8 d

**TABLE 4:** Plackett-Burman experimental design matrix and results.

Serial number	Factors								Production
	X_1_	X_2_	X_3_	X_4_	X_5_	X_6_	X_7_	X_8_	
1	−1	1	−1	−1	−1	1	1	1	339.05
2	−1	1	1	−1	1	−1	−1	−1	217.18
3	1	1	−1	1	−1	−1	−1	1	321.31
4	1	−1	1	−1	−1	−1	1	1	362.99
5	1	1	1	−1	1	1	−1	1	220.18
6	−1	−1	−1	1	1	1	−1	1	182.30
7	−1	−1	1	1	1	−1	1	1	178.28
8	1	−1	−1	−1	1	1	1	−1	210.97
9	−1	−1	−1	−1	−1	−1	−1	−1	373.12
10	1	1	−1	1	1	−1	1	−1	190.10
11	−1	1	1	1	−1	1	1	−1	349.05
12	1	−1	1	1	−1	1	−1	−1	327.34

Using ectoine titer as the response variable, regression analysis was performed with Minitab 21 to establish a first-order regression equation:


Y=272.66-0.51X+10.16X+23.18X-314.59X-472.82

X-51.17X-60.92X-75.30X.8

The coefficient of determination (*R*^2^) for the model was 98.96%, indicating excellent goodness-of-fit, and thus the equation is suitable for predicting ectoine titer. Regression analysis identified the statistical significance of the eight factors on the response variable. Among the tested factors, complex salt concentration (*P* = 0.045) and medium fill volume (*P* = 0.000) exhibited *P* < 0.05 (Table 5), confirming their statistically significant impacts on ectoine titer, with medium fill volume showing the most pronounced effect. The remaining six factors had no significant influence. The *T*-values for medium fill volume (−16.52) and complex salt concentration (−3.31) indicated negative correlations between these factors and ectoine production.

**TABLE 5:** Main effects analysis of Plackett-Burman design.

Factors	Coefficient	*T*	*P*
Constant	272.66	61.87	0.000
Sucrose	−0.515	−0.12	0.916
Soybean peptone	0.16	0.04	0.974
Sodium glutamate	3.18	0.72	0.523
Complex salt concentration	−14.59	−3.31	0.045
Medium fill volume	−72.82	−16.52	0.000
Temperature	−1.17	−0.27	0.807
Inoculation volume	−0.92	−0.21	0.849
Fermentation time	−5.30	−1.20	0.315

#### 3.3.2 Steepest ascent experiment

The Plackett-Burman design revealed that complex salt concentration and medium fill volume were the critical factors influencing ectoine production in TRM83610. Both factors exhibited negative effects, necessitating appropriate reduction of their values. Step sizes were determined based on the magnitude of these factors: medium fill volume was incrementally decreased starting from 115 mL, and complex salt concentration was reduced stepwise from 125 g/L. Other conditions were set to the optimized values determined by single-factor experiments. After 6 days of fermentation, ectoine content in the biomass was quantified. Group 3 achieved the maximum ectoine titer (Table 6). Consequently, the factor levels from Group 3 (complex salt concentration: 109 g/L; medium fill volume: 85 mL) were selected as the central point for subsequent response surface optimization design.

**TABLE 6:** Design of the steepest ascent experiment.

Run	Medium fill volume (ml)	Complex salt concentration (g/L)	Titer (mg/L)
1	115	125	294.54
2	100	117	325.04
3	85	109	427.00
4	70	101	386.61
5	55	93	311.50

#### 3.3.3 Response surface design

A central composite design (CCD) was implemented using Minitab 21 software to investigate the effects of complex salt concentration and medium fill volume on ectoine titer. Ectoine production (Y) was designated as the response variable, with complex salt concentration (X*4*) and medium fill volume (X*5*) as independent variables. Each factor was tested at five coded levels (−1.414, −1, 0, 1, 1.414). The factors and levels for the CCD are detailed in Table 7. Results from the CCD indicated that the fermentation conditions of 109 g/L complex salt concentration and 85 mL medium fill volume approached optimality for ectoine production (Table 8).

**TABLE 7:** Factors and levels of the central composite design (CCD) in response surface methodology.

ID	Name	Level				
		−1.414	−1	0	1	1.414
X_4_	Complex salt concentration (g/L)	97.68	101.00	109.00	117.00	120.31
X_5_	Medium fill volume (mL)	63.79	70.00	85.00	100.00	106.21

**TABLE 8:** Results of the central composite design (CCD).

Run	Complex salt concentration (g/L)	Medium fill volume (mL)	Titer (mg/L)
1	117.00	70.00	412.29
2	101.00	100.00	370.93
3	109.00	106.21	359.54
4	109.00	85.00	443.16
5	101.00	70.00	397.66
6	120.31	85.00	417.71
7	97.68	85.00	410.20
8	109.00	85.00	447.29
9	117.00	100.00	386.61
10	109.00	85.00	451.58
11	109.00	85.00	459.87

A quadratic polynomial regression equation was derived via binary regression fitting in Minitab 21, expressing ectoine titer (Y) as a function of complex salt concentration (X*4*) and medium fill volume (X_5_) in uncoded units:


Y=447.54+0.638X4-0.713X5-0.2651X4-20.1704

X5+20.0022X4×X5

Analysis of variance (ANOVA) demonstrated a high model reliability, with an *F*-value of 41.87 and a *P*-value of 0.000.

#### 3.3.4 Response surface analysis and validation

**FIGURE 12:**
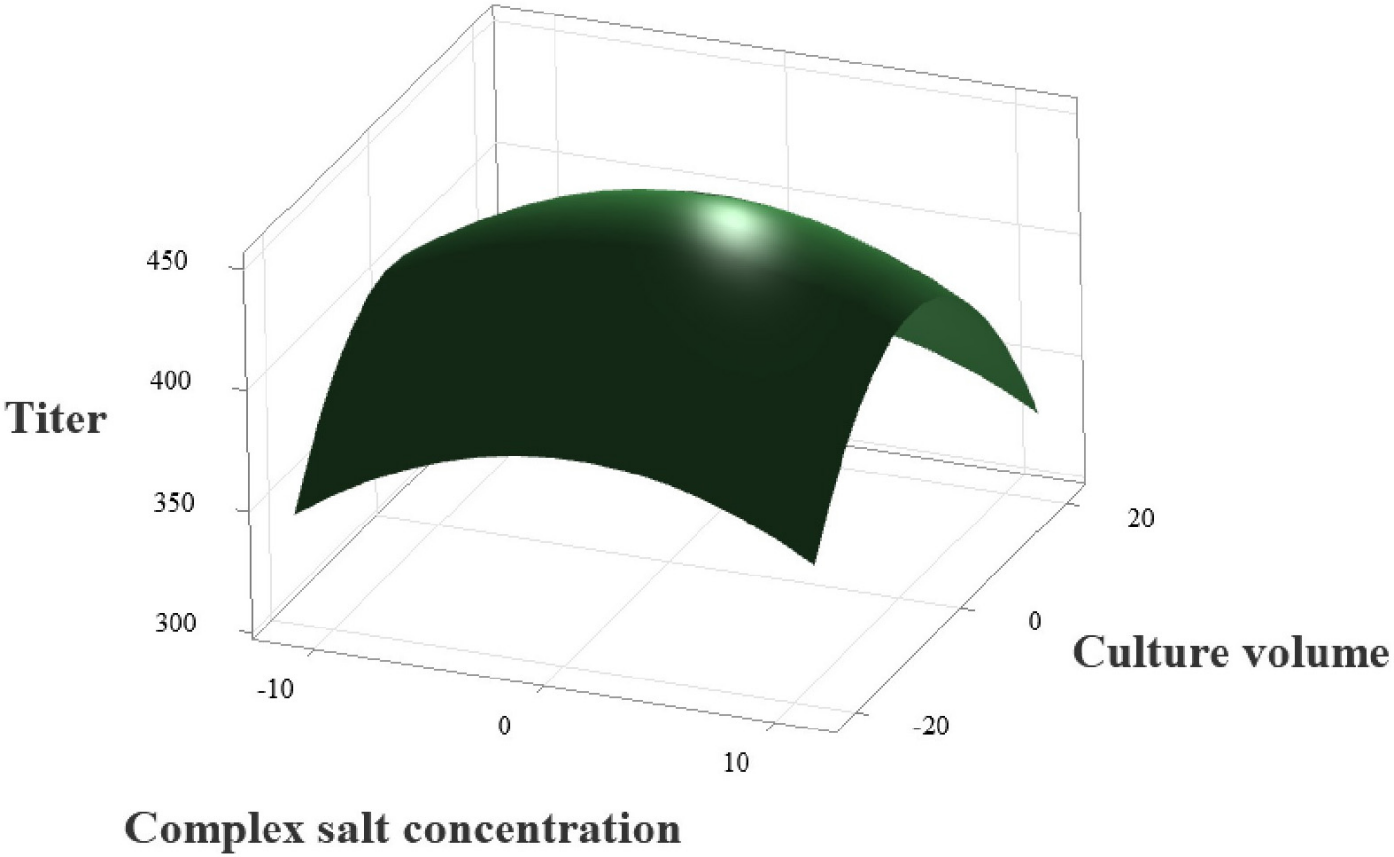
Response surface plot of complex salt concentration and medium fill volume on ectoine titer.

**FIGURE 13:**
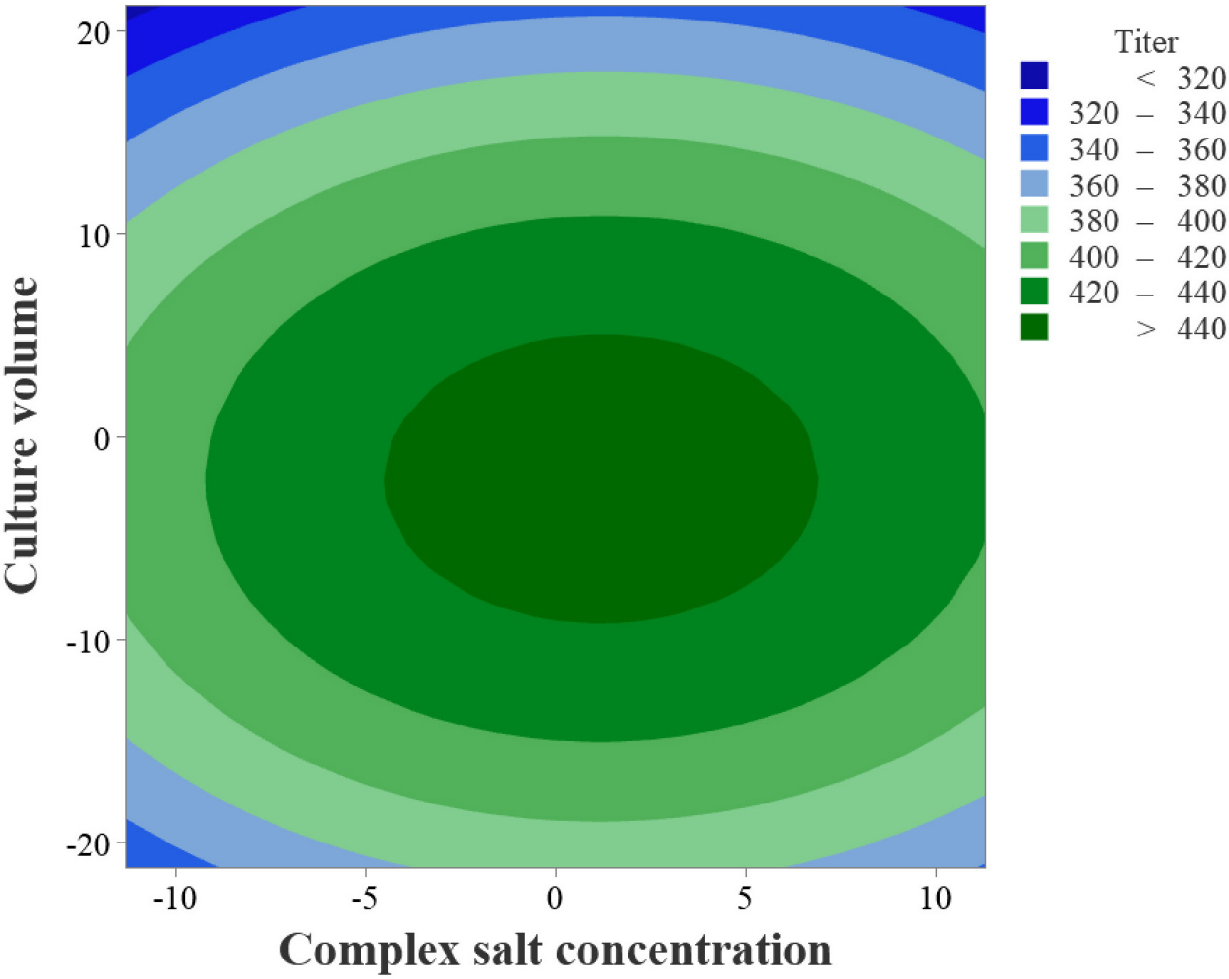
Contour plot of complex salt concentration and medium fill volume on ectoine titer.

Response surface plots (Figure 12) and contour plots (Figure 13) were generated using Minitab 21 software. As shown in Figure 11, the downward-opening response surface indicates the presence of a maximum point in the regression model. By solving the first-order partial derivatives of the regression equation, the predicted maximum ectoine titer of 448.66 mg/L was identified at 110.19 g/L complex salt concentration (X_4_) and 82.92 mL medium fill volume (X_5_). Balancing model predictions and practical feasibility, the optimized conditions were adjusted to 110.20 g/L complex salt concentration and 83.00 mL medium fill volume.

Validation experiments were conducted using the optimized soybean peptone-sucrose medium and cultivation parameters. Ectoine content in the biomass was quantified via high-performance liquid chromatography (HPLC). The measured ectoine titer reached 440.60 mg/L, closely matching the theoretical prediction (448.66 mg/L) and representing a 6.22-fold increase over the initial titer of 70.75 mg/L.

## 4 Discussion

This study, through metabolomic analysis, detected six compatible solutes predominantly consisting of ectoine in the cells of the wild-type strain *B. epidermidis* TRM83610, among which the presence of Nε-acetyl-L-lysine has been reported for the first time in the genus *Brevibacterium*. Systematic fermentation optimization increased the ectoine titer by 6.22-fold, reaching 440.62 mg/L. Further metabolic network analysis suggested potential strategies employed by the strain in response to salt stress, including multi-layered metabolic adaptations such as osmoadaptation, oxidative stress resistance, and competitiveness for survival. These results indicate that strain TRM83610 is not only a promising ectoine-producing bacterium, but its unique intracellular compatible solute profile and possible multi-dimensional stress response mechanisms also provide new metabolomic evidence for further understanding its environmental adaptation strategies.

Metabolomic analysis identified six compatible solutes, predominantly ectoine, whose concentrations varied significantly with NaCl levels, albeit with distinct trends. Previous studies indicate that halotolerant microorganisms synthesize diverse compatible solutes depending on environmental conditions (Han et al., 2018), dynamically adjusting metabolic pathways to regulate solute concentrations and maintain osmotic balance. A proposed “two-phase” salt tolerance strategy (Czech et al., 2018; Roesser and Muller, 2001), suggests an initial rapid response phase, where cells accumulate readily available solutes (e.g., betaine, L-glutamic acid) to counteract osmotic stress and enable proliferatio (Martin et al., 2001; Robertson et al., 1992), followed by synthesis of more complex solutes like ectoine and Nε-acetyl-L-lysine for long-term protein stabilization. Aston and Peyton (2007) observed salinity-dependent shifts in compatible solute profiles in *Halomonas campisalis*. This phased strategy appears to accurately explain the observations from our study. Although our research lacks dynamic monitoring evidence, we found that the ABC transporter pathway is the most significantly enriched metabolic pathway. This pathway includes the Opu family, ProU family, and the EhuABCD system, which function to rapidly transport compatible solutes such as glycine betaine and ectoine from the external environment into microbial cells (Bremer and Krämer, 2019). For metabolomic analysis, bacterial cells were obtained from a medium containing yeast extract, which contains a small amount of glycine betaine. The ABC transporters may transport this into *B. epidermis* cells to counteract osmotic stress. Therefore, we propose that *B. epidermis* TRM83610 initially accumulates betaine, L-glutamic acid, L-glutamine, and L-proline as temporary osmoprotectants during early NaCl stress, later transitioning to ectoine as the primary solute. Notably, aspartate, glutamate, and glutamine directly participate in ectoine biosynthesis, whereas betaine and proline may indirectly support its synthesis. Their declining concentrations, inversely correlated with ectoine accumulation, suggest metabolic reprogramming under salt stress (Patel et al., 2018; Schwendner et al., 2018). However, these conjectures and hypotheses require further investigation.

Correlation analysis revealed strong negative associations between ectoine and 14 organic acids/derivatives. While amino acid consumption has been linked to ectoine synthesis (Sevin et al., 2016; Shu et al., 2023; Zou et al., 2024), this study implicates non-amino organic acids in this process. Intriguingly, Nε-acetyl-L-lysine showed no significant correlation with other solutes and decreased sharply at 15% NaCl, suggesting roles beyond osmoprotection, potentially in protein acetylation to enhance stress resistance (Kremer et al., 2024; Stojowska-Swedrzynska et al., 2024).

*B. epidermidis* TRM83610 employs multifaceted NaCl adaptation strategies. Elevated 5-hydroxyindoleacetate (a serotonin oxidation product) under high salinity indicates oxidative stress mitigation (Gres et al., 2013; Planells-Carcel et al., 2025), while upregulated antimicrobial compounds suggest enhanced ecological competitiveness (Yovchevska et al., 2025; Giddings and Newman, 2022). Metabolic pathway enrichment (e.g., amino acid biosynthesis, D-amino acid metabolism and ABC transporters) highlights coordinated regulation of ectoine synthesis and stress-responsive pathways. Antimicrobial/antioxidant metabolites, though distributed across pathways, predominantly localized to “Metabolic pathways” and “Biosynthesis of plant secondary metabolites,” underscoring the strain’s integrated osmotic, oxidative, and competitive adaptation mechanisms.

This study provides a preliminary evaluation of the application potential of *B. epidermidis* TRM83610. Firstly, the strain exhibits strong ectoine synthesis capability. Wild-type strains typically demonstrate low ectoine titers, generally not exceeding 270 mg/L (Cho et al., 2022; Zhang et al., 2022). For instance, Hong Y. et al. (2019) isolated two halophilic strains from Jilantai Salt Lake soil with maximum ectoine titers of 80.35 mg/L and 97.89 mg/L, respectively. Yao and Gu (2017) reported an ectoine titer of 92.41 mg/L for *Halomonas ventosae* Al12T AY268080. Wang et al. (2025) obtained a *Halomonas campaniensis* XH26 mutant via UV-induced mutagenesis, achieving ectoine titers ranging from 260 to 1,500 mg/L. In this study, fermentation optimization of the wild-type *B. epidermidis* TRM 83610 resulted in a significantly higher ectoine titer of 440.62 mg/L, representing a 6.22-fold increase over the initial titer of 70.75 mg/L, demonstrating its robust ectoine production capacity (Table 9).

**TABLE 9:** A comparison of ectoine yield and productivity in several microorganisms.

Strain	Culture time (h)	Titer (mg)	Productivity (mg/h/L)	Substrate-to-product yield (mg/g)	Growth conditions	References
*B. epidermidis* TRM 83610 (wild-type strain)	144	440.62	3.06	43.42	34°C, 110.2 g/LNaCl	This study
Methylomicrobium alcaliphilum 20ZDP (Wild-type strain)	96	142.32	1.48	3.36	30°C, 60 g/L NaCl	(Cho et al., 2022)
Streptomyces pactum JG9 (Wild-type strain)	72	97.89	1.36	39.15	28°C, 50 g/L NaCl	(Hong Y. et al., 2019)
*Halomonas* sp. W2 (Wild-type strain)	36	92.41	2.57	46.21	35°C, 100 g/L NaCl	(Yao and Gu, 2017)
Halomonas campaniensis XH26 (wild-type strain)	30	260	8.7	40	35°C, 60 g/L NaCl	(Wang et al., 2025)
Halomonas campaniensis G8-52 (UV-mutated strain)	28	1500.00	53.57	300	35°C, 87 g/L NaCl	(Wang et al., 2025)

The synthesis of exotoxins is influenced by multiple factors, among which the combination and concentrations of carbon and nitrogen sources, pH, temperature, salinity, and agitation rate exert considerable influence. Studies have shown that yeast extract yields the highest ectoine titer compared to other tested carbon sources, including glucose, glutamate, fructose, starch, and sucrose (Chen et al., 2018). Another study reported that sucrose induced the highest intracellular ectoine accumulation in *Nesterenkonia xinjiangensis*, followed by mannitol, glucose, lactose, and maltose (Orhan et al., 2023). In the present study, we found sucrose to be superior to other tested carbon sources, including maltose, glucose, corn flour, oats, millet, and starch.

Regarding nitrogen source screening, while some studies have confirmed inorganic nitrogen sources as the most effective (Orhan et al., 2023; Lim et al., 2024), others have demonstrated the superiority of organic nitrogen sources (Joghee and Jayaraman, 2016). Notably, several studies indicate that yeast extract is the most effective nitrogen source for ectoine synthesis among those tested. Conversely, some research has shown that complex nitrogen sources, such as tryptone, soy peptone, peptone, and casamino acids, significantly promote ectoine production, with tryptone being particularly effective. The same study also confirmed that ammonium acetate could replace these as the most efficient nitrogen source (Wei et al., 2011). In this study, we observed that soy peptone performed better than other tested nitrogen sources, including yeast extract, peptone, acid-hydrolyzed casein, fish peptone, beef extract, and ammonium chloride.

These findings collectively indicate that different strains exhibit distinct nitrogen source preferences during ectoine synthesis. This variation may be attributed to the fact that many microorganisms have evolved multiple aspartokinase isoenzymes to meet the demands of specific biosynthetic pathways. These isoenzymes are subject to feedback regulation by downstream end-products (Stöveken et al., 2011). Differences in these enzymes among strains likely contribute to the observed variations in how different carbon and nitrogen sources influence ectoine synthesis.

When the environmental pH deviates by one unit (either lower or higher) from the optimal pH for a microorganism, its growth rate and metabolic activity can decrease by up to 50% (Ye et al., 2012). The optimal pH varies significantly among different strains, ranging from acidic conditions, such as pH 5.90 for *Halomonas* sp. (Li et al., 2017), to alkaline conditions, such as pH 9.0 for *Alkalibacillus haloalkaliphilus* (Bergmann et al., 2013), and neutral conditions, including pH 7.0 for *Halomonas salina* and *Marinococcus* sp., and pH 7.5 for *Salinivibrio* sp. (Lang et al., 2011, Wei et al., 2011; Omara et al., 2020). Our study confirms that the optimal pH for *Brevibacterium epidermis* is 7.5, indicating a preference for a neutral environment.

Studies have shown that temperatures below 20°C or above 37°C can inhibit ectoine production. Within the range of 25–30°C, ectoine yield increases, reaching a plateau at 30–37°C, and slightly declines at 40°C. Notably, *Marinococcus* sp. ECT1 cannot grow at 45°C (Wei et al., 2011). Our research indicates that a temperature range of 28–37°C is suitable for ectoine synthesis by *B. epidermis* TRM83610, with the optimum at 34°C.

Agitation rate affects dissolved oxygen levels; insufficient agitation fails to meet microbial oxygen demands, while increasing agitation enhances oxygen supply within a certain range. However, excessively high agitation may elevate pH, potentially inhibiting microbial growth (Bergmann et al., 2013). Our findings suggest that an agitation rate between 160 and 200 r/min is suitable for ectoine synthesis by *B. epidermis*, with no significant difference in ectoine titer observed between 180 and 200 r/min. Therefore, we conclude that 180 r/min is sufficient to meet the oxygen demand of this strain, which aligns with previous reports (Wei et al., 2011).

An appropriate salt concentration is a critical stimulus for ectoine synthesis, whereas excessively high salinity can adversely affect cells, including cell wall and membrane integrity, cytoplasmic hydration, and fatty acid synthesis (Wood, 2015). Our study found that a composite salt concentration of 110.2 g/L is optimal for ectoine synthesis by *B. epidermis*. This value is lower than those reported for *Alkalibacillus haloalkaliphilus* (149.0 g/L) (Bergmann et al., 2013) and *Halomonas organivorans* (180.0 g/L) (Van Thuoc et al., 2019), but higher than that for *Salinivibrio costicola* (80.0 g/L) (Omara et al., 2020).

In summary, during ectoine synthesis by *B. epidermis*, a composite salt concentration of 110.2 g/L provides the maximal stimulus for ectoine production, 3 g/L sucrose serves as the carbon source, and 25 g/L soy peptone supplies amino acids. Sodium glutamate at 0.06 mol/L dissociates to provide glutamate ions, which are subsequently converted within the cells into glutamate—a key precursor for ectoine biosynthesis (Omara et al., 2020). Cultivation conditions of 34°C and pH 7.5 ensure maximal activity of enzymes involved in ectoine synthesis, while an agitation rate of 180 r/min supplies the necessary dissolved oxygen for growth and metabolism. The synergistic action of these factors enables *B. epidermis* to achieve an ectoine titer of 440.62 mg/L.

Furthermore, untargeted metabolomics annotated multiple antimicrobial active substances, a survival strategy advantageous for becoming the dominant strain in open fermentation processes (Martinez et al., 2022). It is noteworthy that 5-hydroxyectoine, if present, is frequently co-isolated with ectoine; its absence of detection in this study thereby reduces downstream recovery costs and process complexity (Liu et al., 2021).

Secondly, *B. epidermidis*TRM 83610 shows promise for broader applications. Nε-acetyl-L-lysine has been proposed for biotechnology applications (Liu et al., 2021). The enrichment of plant growth-promoting metabolites suggests its potential as a Plant Growth-Promoting Bacterium (PGPB). Additionally, the possible production of various antimicrobial and anti-inflammatory active substances further expands its application prospects. Finally, it is noteworthy that Azetidomonamide A was annotated as a different metabolite (DM) via machine learning. To our knowledge, this compound has previously only been reported in *Pseudomonas aeruginosa* (Ernst et al., 2022). In this study, the relative abundance of Azetidomonamide A initially increased and subsequently decreased with rising NaCl concentrations. Previous research has demonstrated that Azetidomonamide A participates in regulating biofilm formation and pigment synthesis in *P. aeruginosa*, and its biosynthesis is modulated by quorum sensing (QS) (Hong Z. et al., 2019). Therefore, we hypothesize that *B. epidermidis* TRM 83610 may perceive and respond to environmental changes through a QS mechanism to modulate physiological activities, warranting further investigation.

## 5 Conclusion

We identified six compatible solutes in *B. epidermidis* TRM83610, including Nε-acetyl-L-lysine. Notably, this is the first report of Nε-acetyl-L-lysine within the genus *Brevibacterium*. The strain employs multipronged NaCl adaptation: ectoine-dominated osmotic regulation, antioxidant synthesis, and antimicrobial production. Its high ectoine titer (440.62 mg/L) and metabolic versatility position it as a promising platform for microbial cell factories, with applications spanning biomanufacturing, agriculture, and biomedicine.

## Data Availability

The datasets generated from untargeted metabolomics and targeted metabolomics analyses in this study are available via the Metabolights database (https://www.ebi.ac.uk/metabolights), under accession numbers MTBLS12624 and MTBLS12638, respectively.

## Footnotes

1
https://www.genescloud.cn

2
https://www.iodraw.com/
