# Supplementary material for: Metabolic response of Brevibacterium epidermidis TRM83610 to NaCl stress
Source: Front Microbiol. 2026 Feb 6;17:1754185. doi: 10.3389/fmicb.2026.1754185 (PMC12920567; doi:10.3389/fmicb.2026.1754185)
Supplement: Supplementary file 1 [file Supplementary_file_1.zip › Supplementary material/Fig S3_Standard curves .docx]

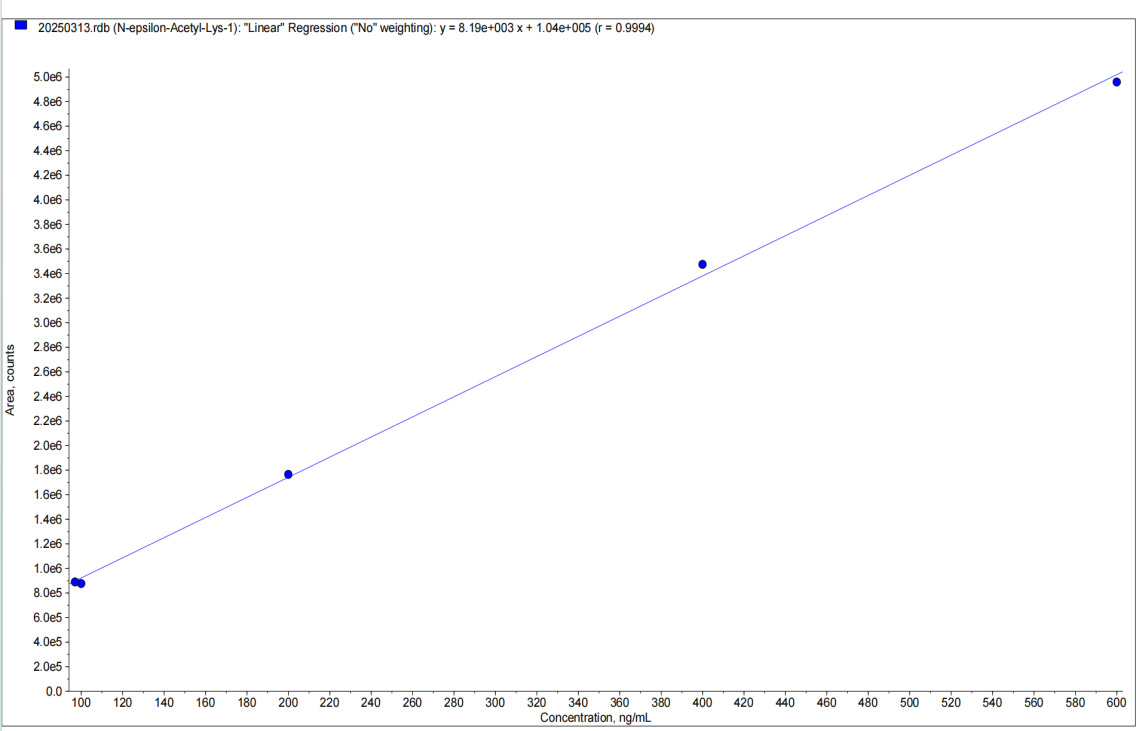


a


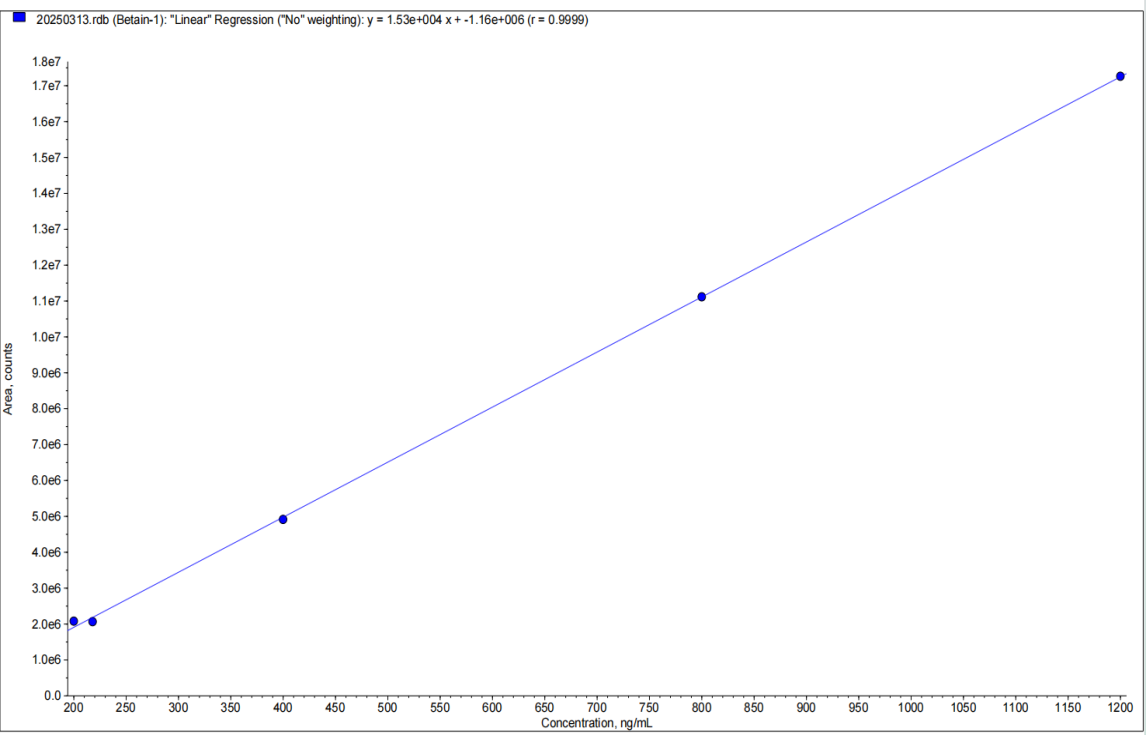


b


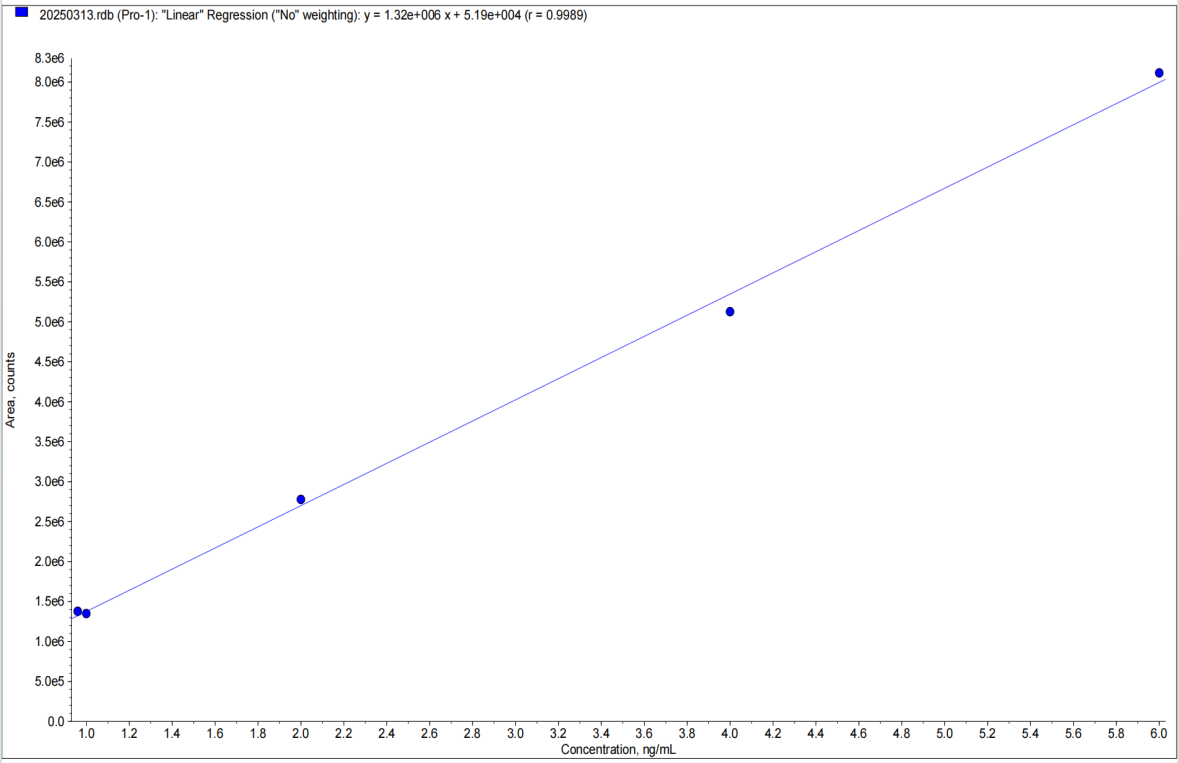


c


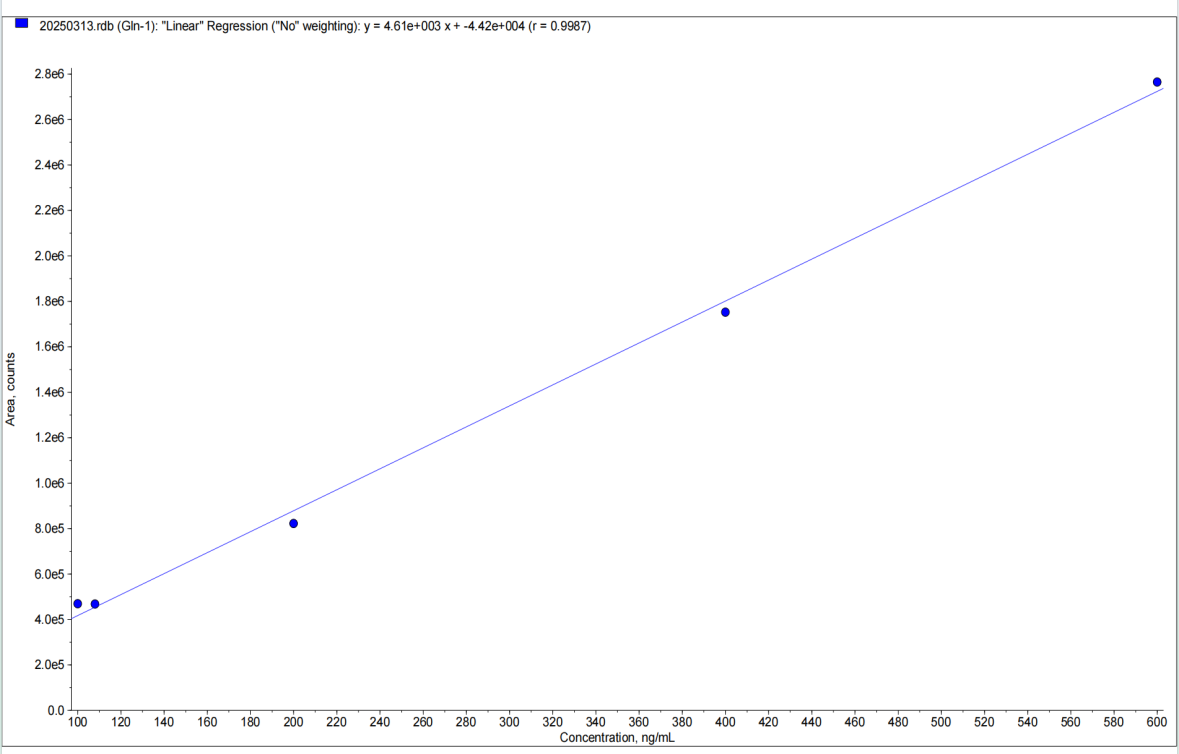


d


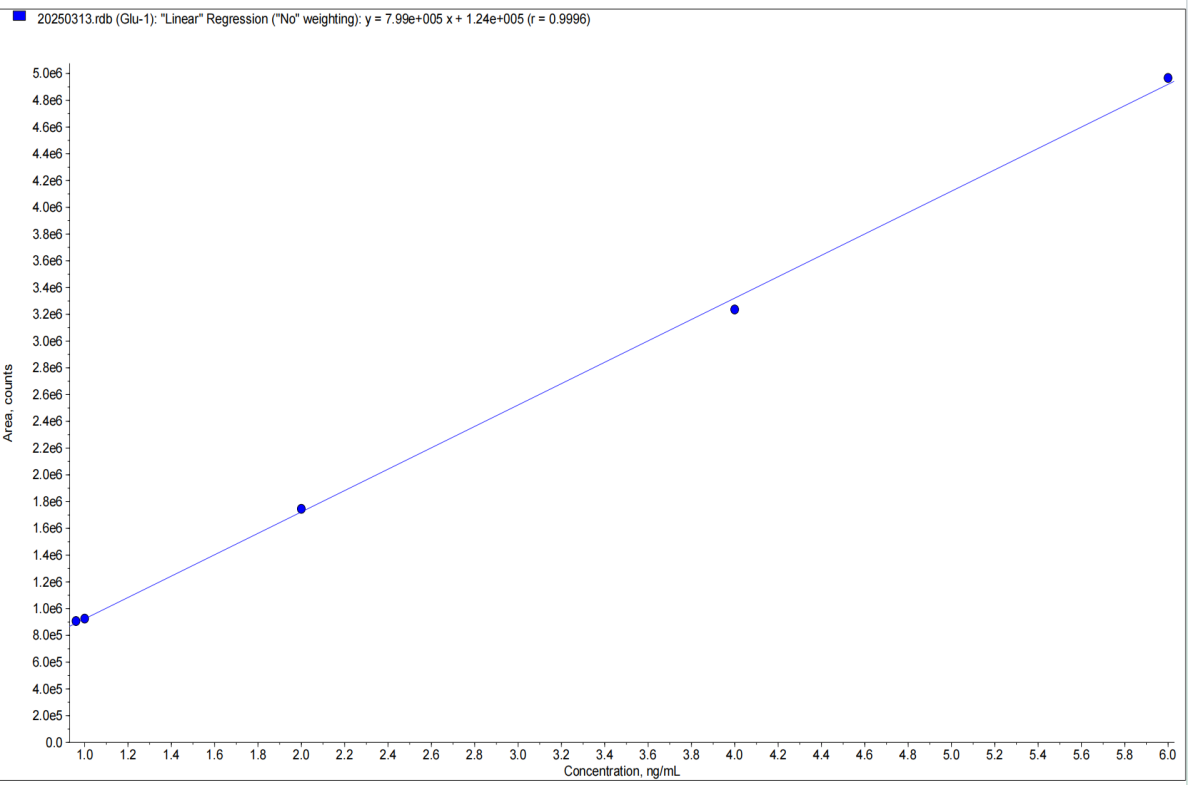


e

**Fig S3 Standard curves for five compatible solutes.** a: Standard curve of Nε-Acetyl-L-lysine; b: Standard curve of Betaine; c: Standard curve of L-Proline; d: Standard curve of L-Glutamine;

e: Standard curve of L-Glutamic acid.
