# Supplementary material for: Metabolic response of Brevibacterium epidermidis TRM83610 to NaCl stress
Source: Front Microbiol. 2026 Feb 6;17:1754185. doi: 10.3389/fmicb.2026.1754185 (PMC12920567; doi:10.3389/fmicb.2026.1754185)
Supplement: Supplementary file 1 [file Supplementary_file_1.zip › Supplementary material/Fig S3_Standard curves .pdf]

a

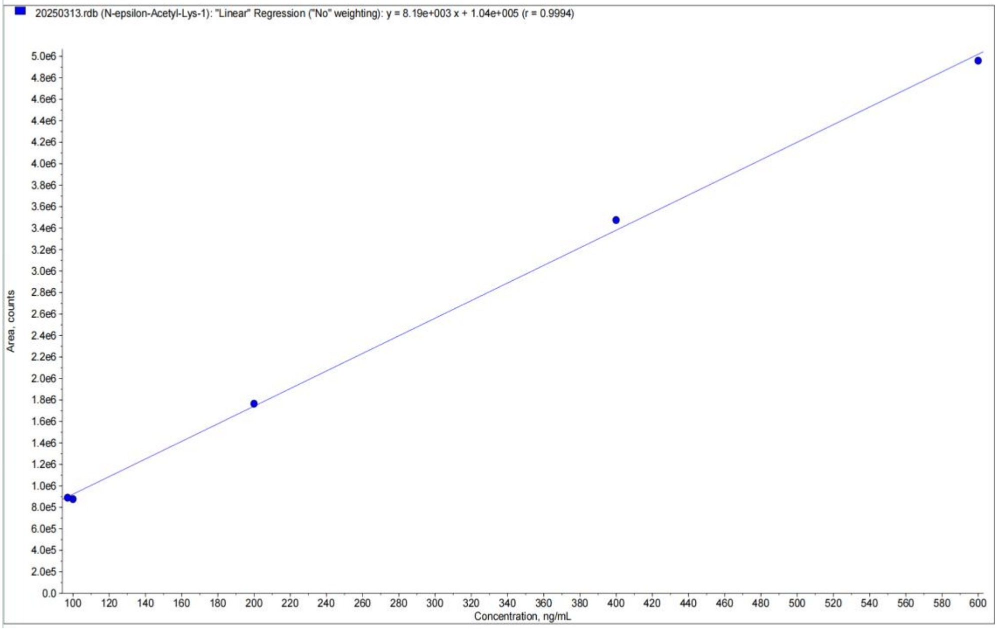
20250313.rdb (N-epsilon-Acetyl-Lys-1): "Linear" Regression ("No" weighting): y = 8.19e+003 x + 1.04e+005 (r = 0.9994)
Area, counts
5.0e6
4.8e6
4.6e6
4.4e6
4.2e6
4.0e6
3.8e6
3.6e6
3.4e6
3.2e6
3.0e6
2.8e6
2.6e6
2.4e6
2.2e6
2.0e6
1.8e6
1.6e6
1.4e6
1.2e6
1.0e6
8.0e5
6.0e5
4.0e5
2.0e5
0.0
100 120 140 160 180 200 220 240 260 280 300 320 340 360 380 400 420 440 460 480 500 520 540 560 580 600
Concentration, ng/mL

b

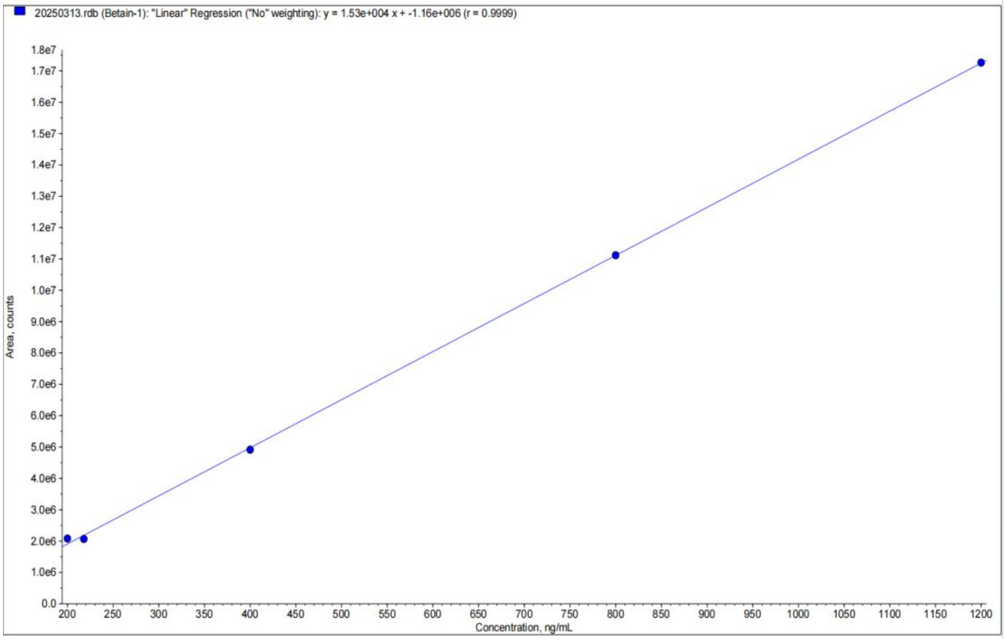
20250313.rdb (Betain-1): "Linear" Regression ("No" weighting): y = 1.53e+004 x + -1.16e+006 (r = 0.9999)
Area, counts
1.8e7
1.7e7
1.6e7
1.5e7
1.4e7
1.3e7
1.2e7
1.1e7
1.0e7
9.0e6
8.0e6
7.0e6
6.0e6
5.0e6
4.0e6
3.0e6
2.0e6
1.0e6
0.0
200 250 300 350 400 450 500 550 600 650 700 750 800 850 900 950 1000 1050 1100 1150 1200
Concentration, ng/mL

c

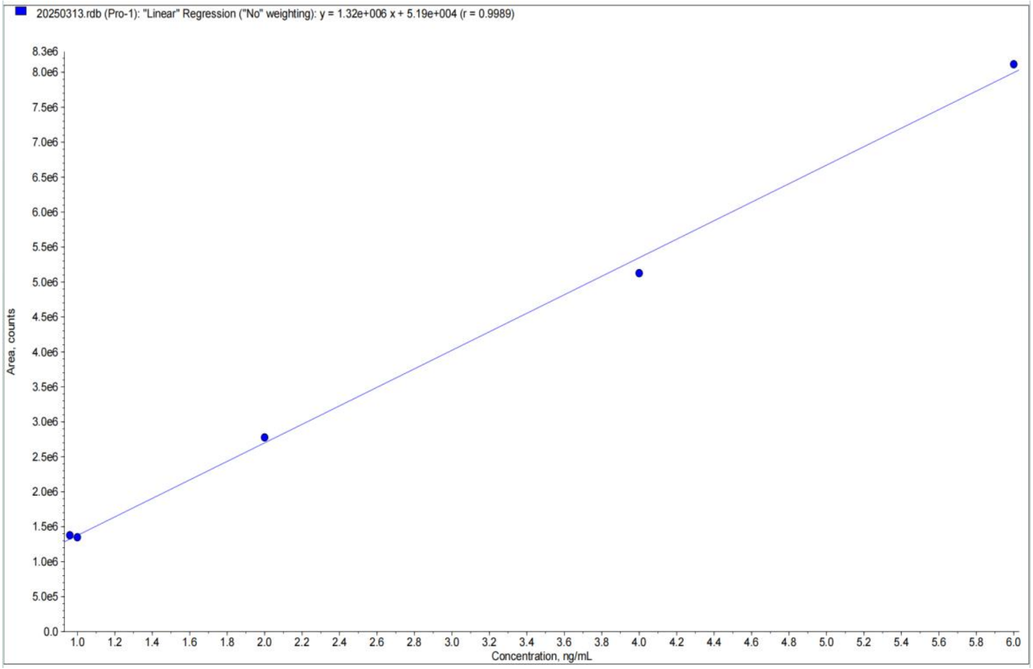

20250313.rdb (Pro-1): "Linear" Regression ("No" weighting): y = 1.32e+006 x + 5.19e+004 (r = 0.9989)
Area, counts
Concentration, ng/mL

d

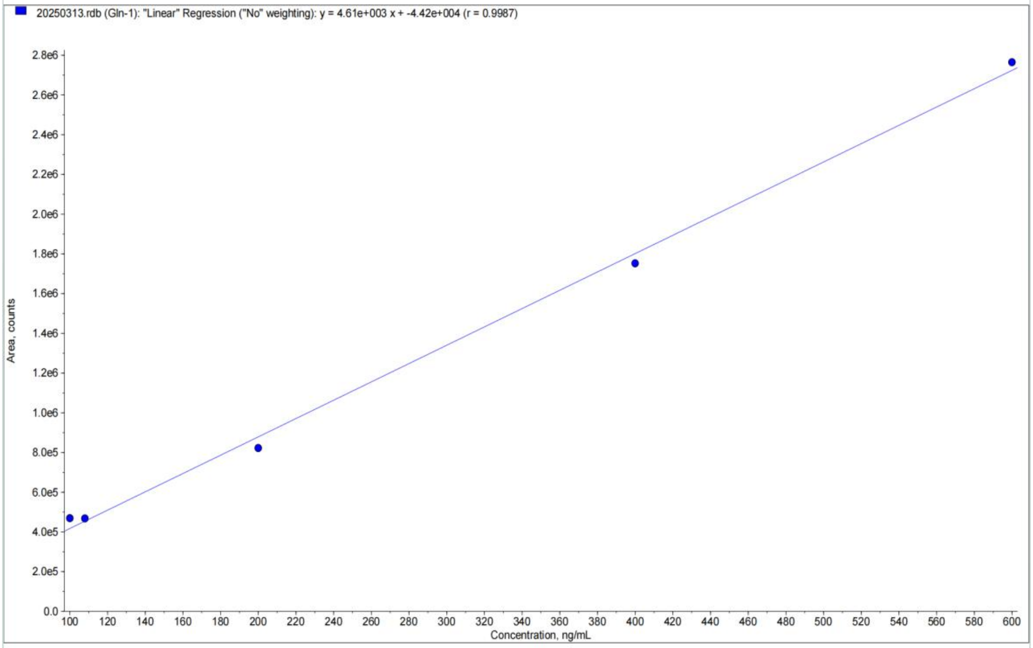

20250313.rdb (Gln-1): "Linear" Regression ("No" weighting): y = 4.61e+003 x + -4.42e+004 (r = 0.9987)
Area, counts
Concentration, ng/mL

e

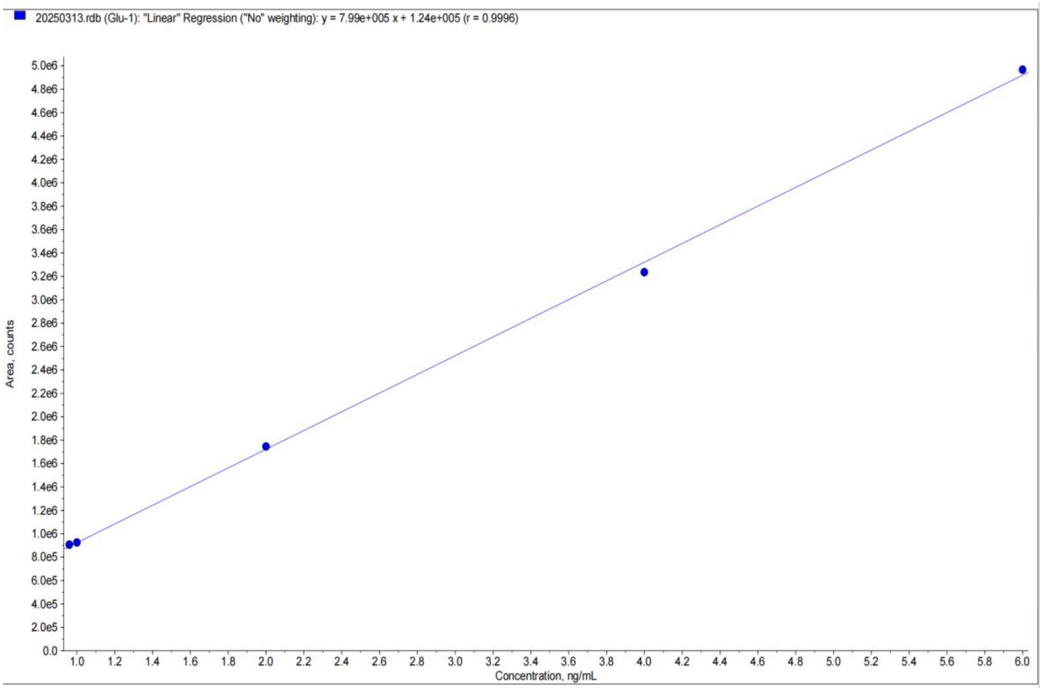

Fig S3　Standard curves for five compatible solutes. a: Standard curve of Nε-Acetyl-L-lysine; b: Standard curve of Betaine; c: Standard curve of L-Proline; d: Standard curve of L-Glutamine; e: Standard curve of L-Glutamic acid.
