# Supplementary material for: Metabolic response of Brevibacterium epidermidis TRM83610 to NaCl stress
Source: Front Microbiol. 2026 Feb 6;17:1754185. doi: 10.3389/fmicb.2026.1754185 (PMC12920567; doi:10.3389/fmicb.2026.1754185)
Supplement: Supplementary file 1 [file Supplementary_file_1.zip › Supplementary material/Fig_S1_OPLS-DA.docx]

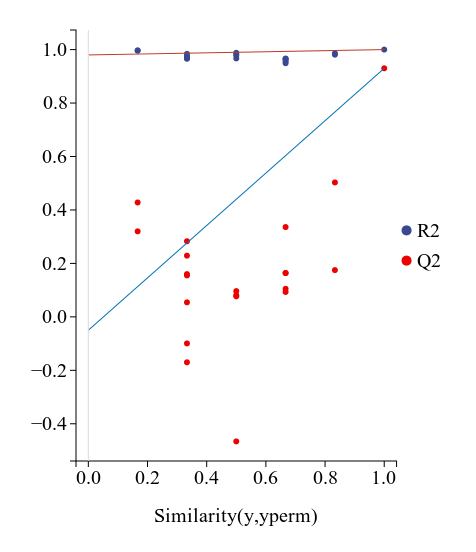

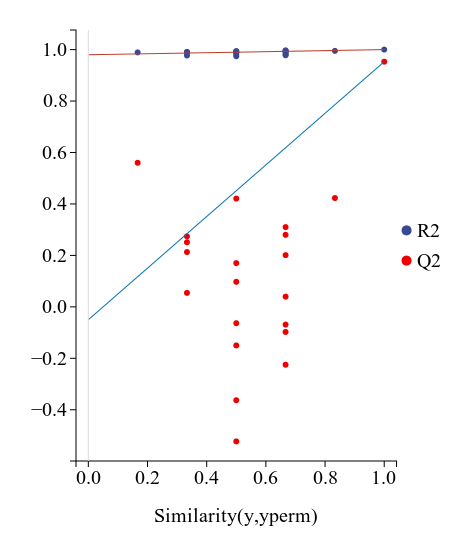

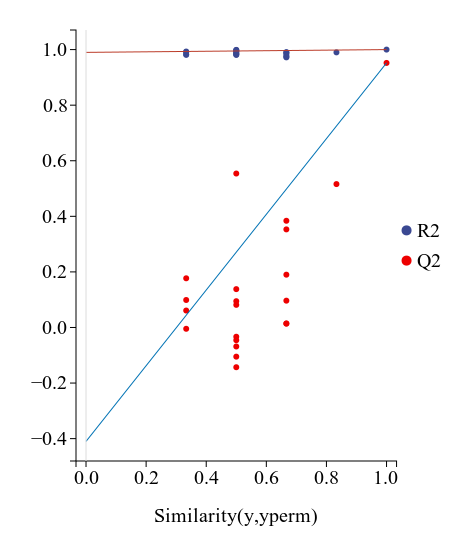


a


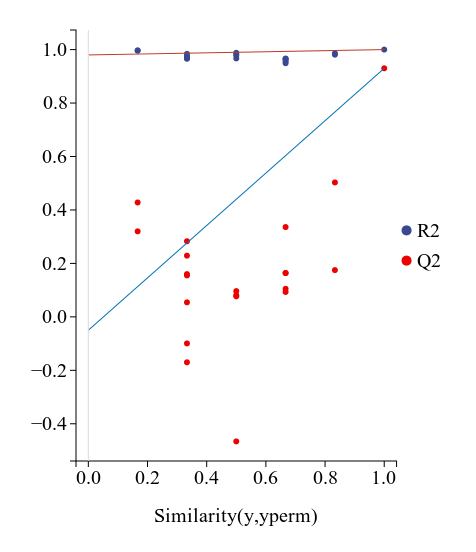

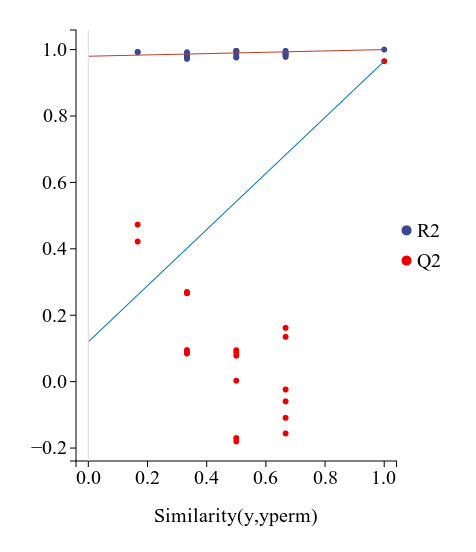

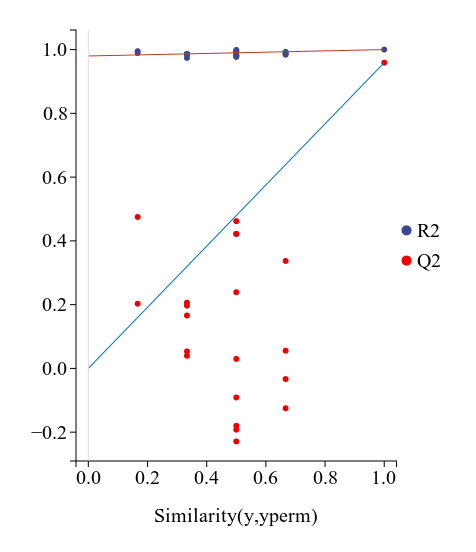


b

**Fig S1 Permutation test plots for OPLS-DA.** a: Permutation test in positive ion mode; b: Permutation test in negative ion mode. From left to right: A vs B, A vs C, A vs D.
