# Supplementary material for: Metabolic response of Brevibacterium epidermidis TRM83610 to NaCl stress
Source: Front Microbiol. 2026 Feb 6;17:1754185. doi: 10.3389/fmicb.2026.1754185 (PMC12920567; doi:10.3389/fmicb.2026.1754185)
Supplement: Supplementary file 1 [file Supplementary_file_1.zip › Supplementary material/Table_S2_Functional DMs.docx]

**Table S2 Significantly enriched functional DMs**

|  | **Biological function** | **Regulation**  **(Group A as control)** | | | **Class** | **References** |
| --- | --- | --- | --- | --- | --- | --- |
|  |  | **B** | **C** | **D** |  |  |
| 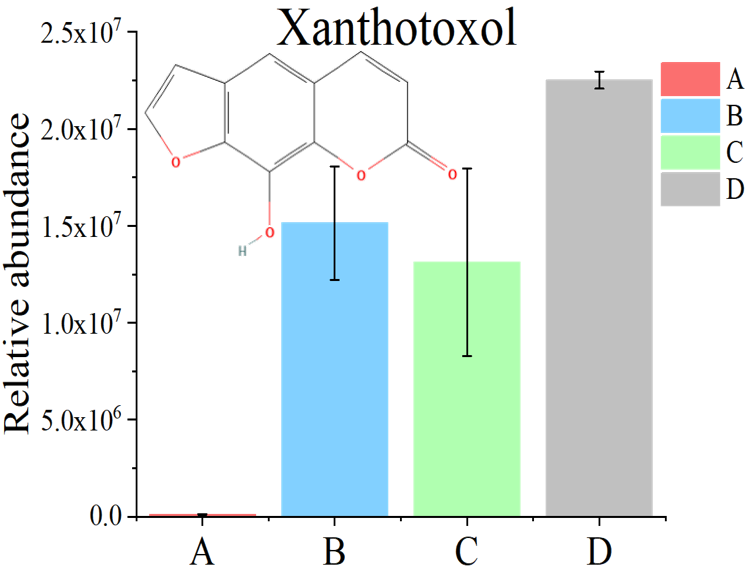 | Antioxidan | Up | Up | Up | Coumarin derivative | (Zhu et al., 2023) |
| 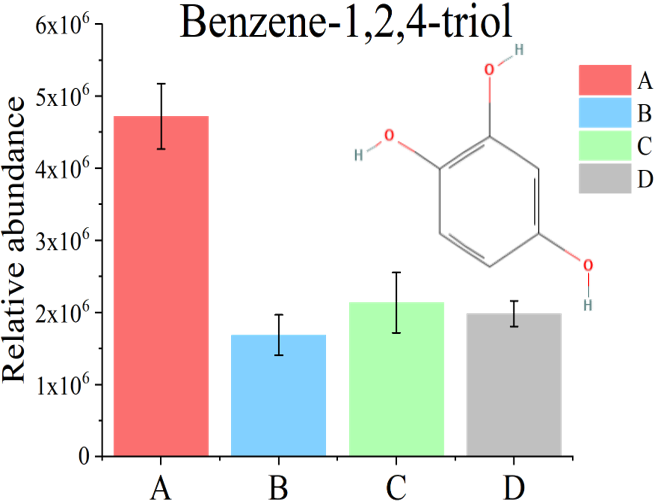 | Antioxidant Antibacterial | Down | Down | Down | Phenol derivative | (Cavalca et al., 2024) |
| 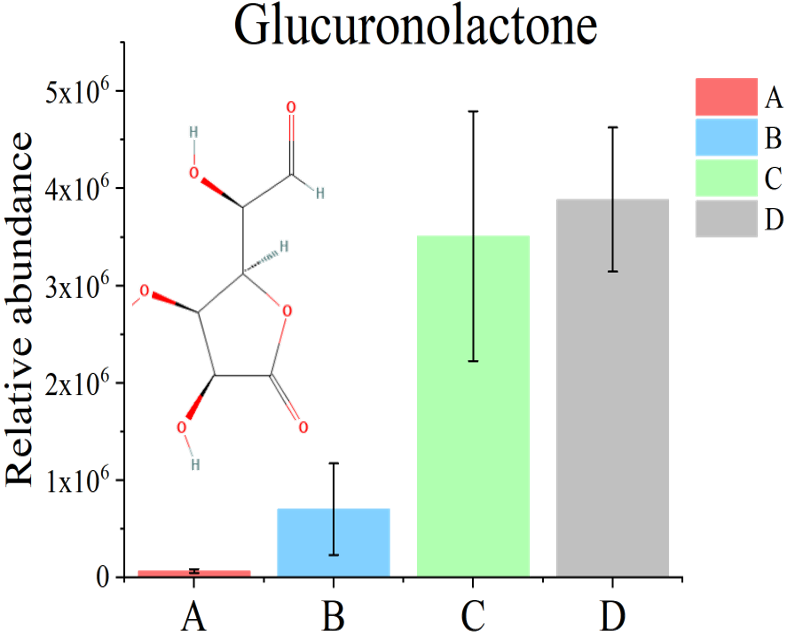 | Antioxidant | Up | Up | Up | Lactone | (Zhang et al., 2025) |
| 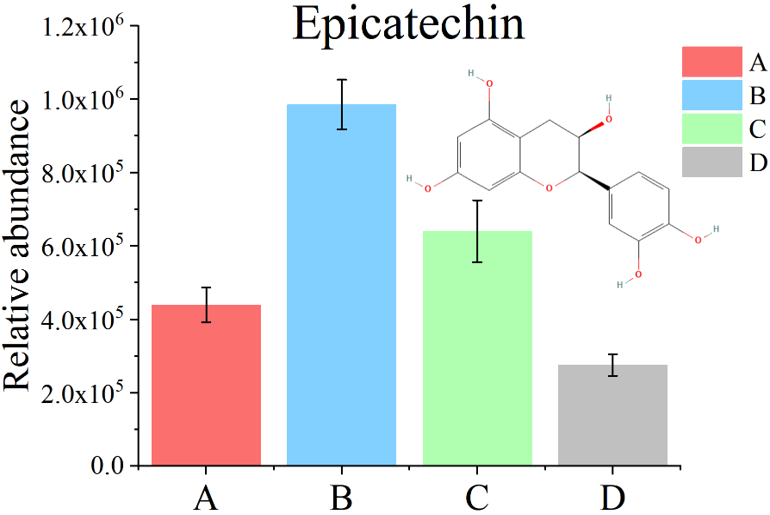 | Yeast inhibitor, Antioxidant | Up | Up | Down | Flavonoid | (Kimani et al., 2021, Zhang et al., 2022) |
| 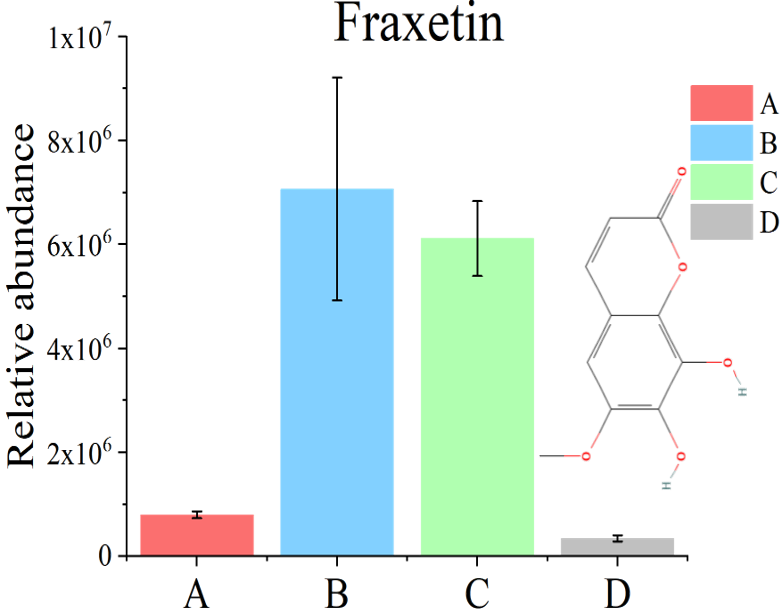 | Antioxidant, Anti-inflammatory, Antibacterial | Up | Up | Down | Coumarin derivative | (Sun et al., 2023, Montagner et al., 2008) |
| 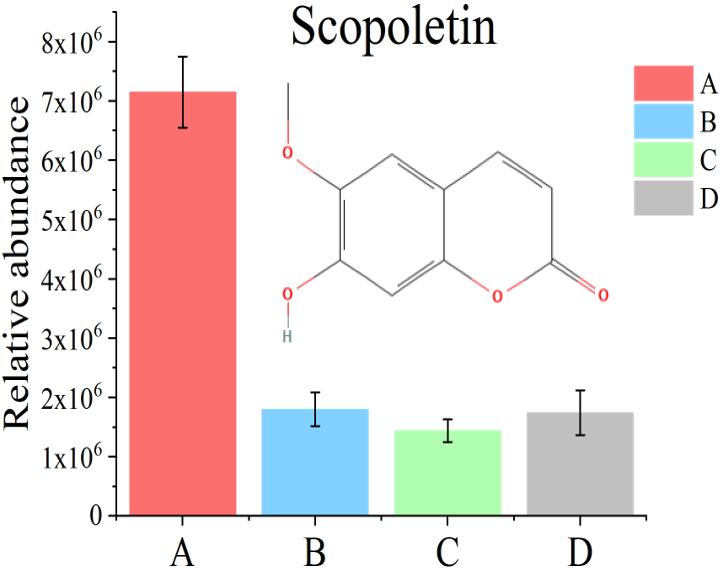 | Antibacterial, Antioxidant | Down | Down | Down | Coumarin derivative | (Antika et al., 2022, Gao et al., 2024, Skroza et al., 2022) |

**Continued table S2 Significantly enriched functional DMs**

|  | **Biological function** | **Regulation**  **(Group A as control)** | | | **Class** | **References** |
| --- | --- | --- | --- | --- | --- | --- |
|  |  | **B** | **C** | **D** |  |  |
| 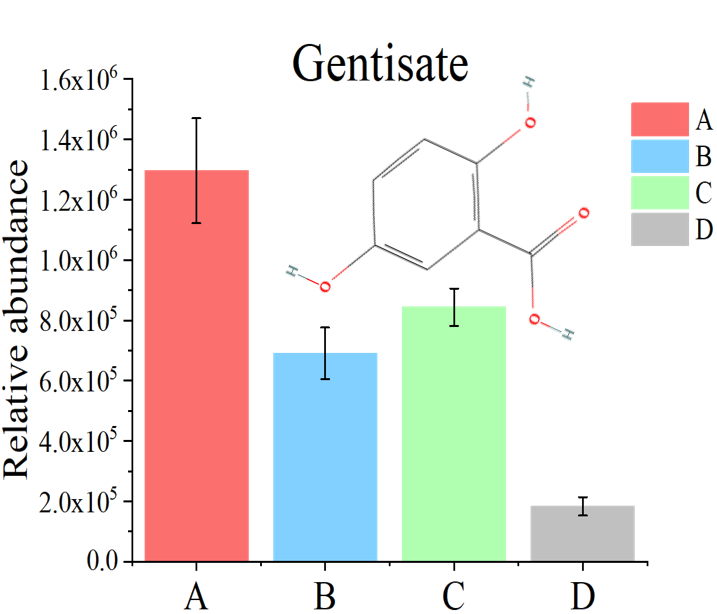 | Antioxidant, Anti-inflammatory | Down | Down | Down | Phenolic acid | (Skroza et al., 2022, Kang et al., 2021) |
| 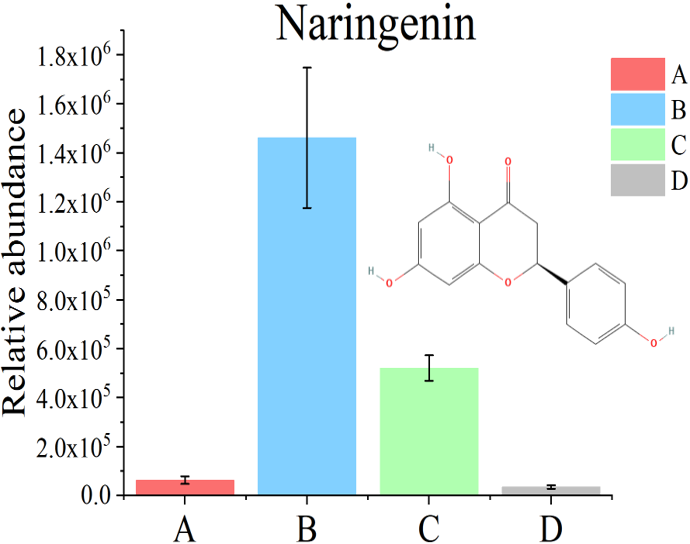 | Antioxidant, Anti-inflammatory | Up | Up | Nodiff | Flavonoid | (Kang et al., 2021) |
| 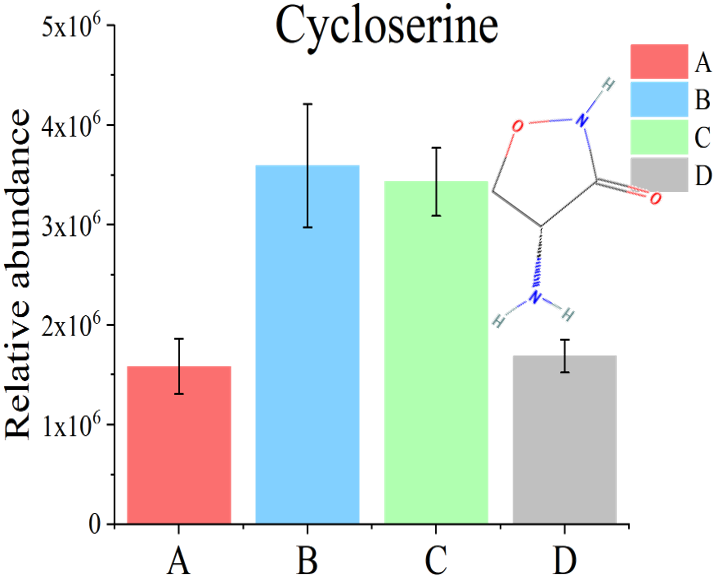 | Antibiotic, Inhibits bacterial cell wall synthesis | Up | Up | Nodiff | Cyclic amino acid | (Chauhan et al., 2024, Robbins et al., 2023) |
| 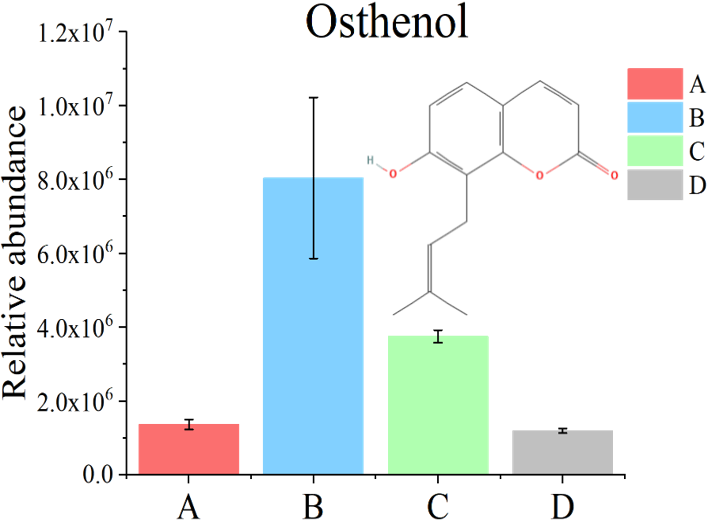 | Anti-inflammatory, Antibacterial or Anticancer | Up | Up | Nodiff | Coumarin derivative | (Montagner et al., 2008) |
| 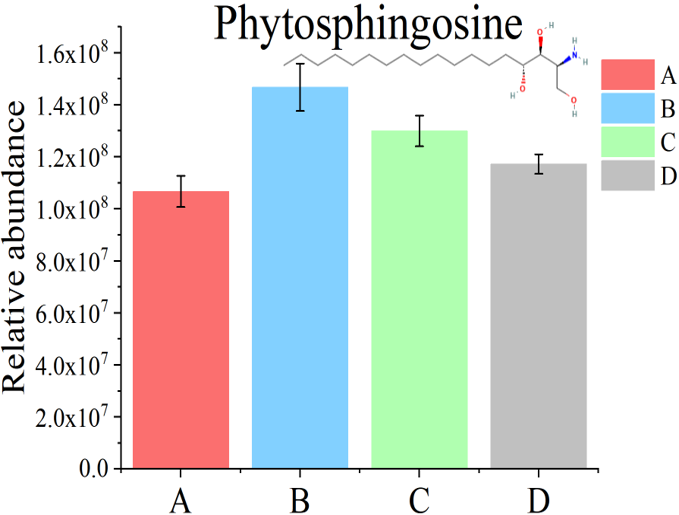 | Antibacterial, Anti-inflammatory | Up | Up | Nodiff | Sphingolipid | (Pejon et al., 2023, Sung et al., 2024) |
| 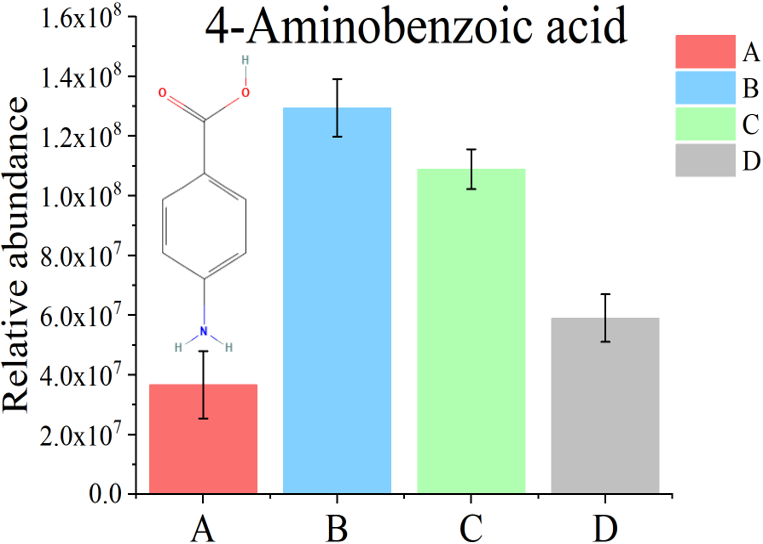 | Antibacterial | Up | Up | Nodiff | Aminobenzoic acid | (Kratky et al., 2019) |

**Continued table 2 Significantly enriched functional DMs**

|  | **Biological function** | **Regulation**  **(Group A as control)** | | | **Class** | **References** |
| --- | --- | --- | --- | --- | --- | --- |
|  |  | **B** | **C** | **D** |  |  |
| 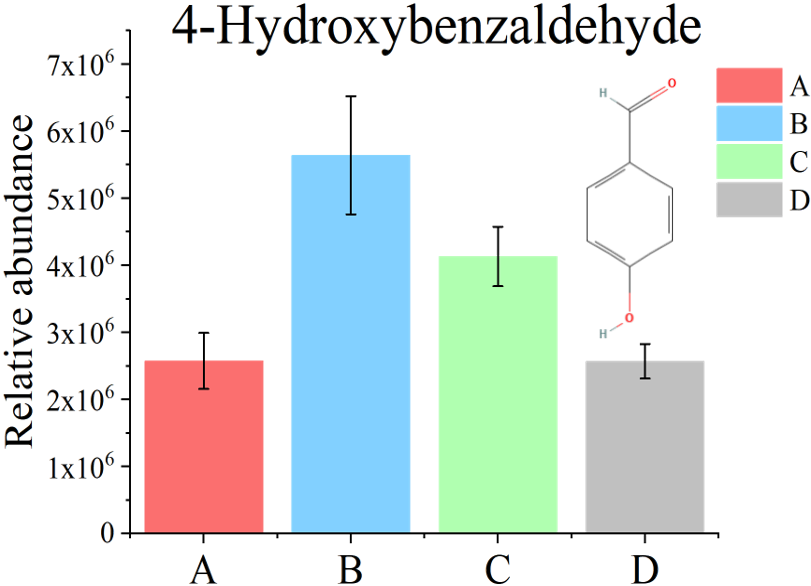 | Antibacterial | Up | Up | Nodiff | Phenolic aldehyde | (Kang et al., 2017, Lee et al., 2020) |
| 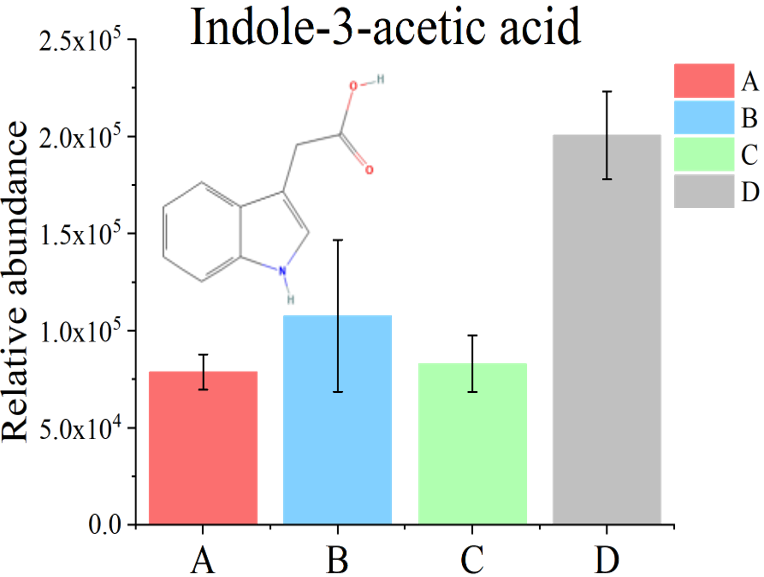 | Plant growth hormone (Auxin), regulates cell elongation and differentiation | Up | Up | Up | Indole derivative | (Khalil et al., 2024) |
| 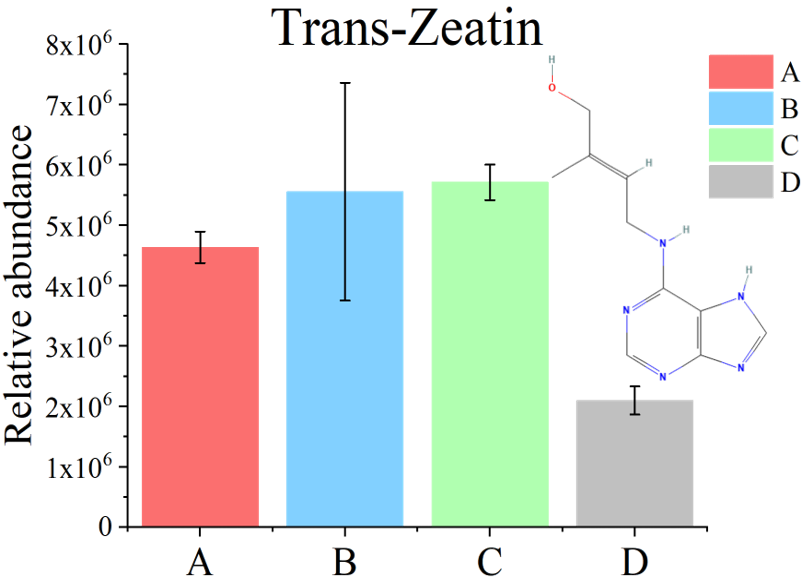 | Plant hormone, promotes cell division | Up | Up | Down | Adenine derivative | (Kiba et al., 2023) |
| 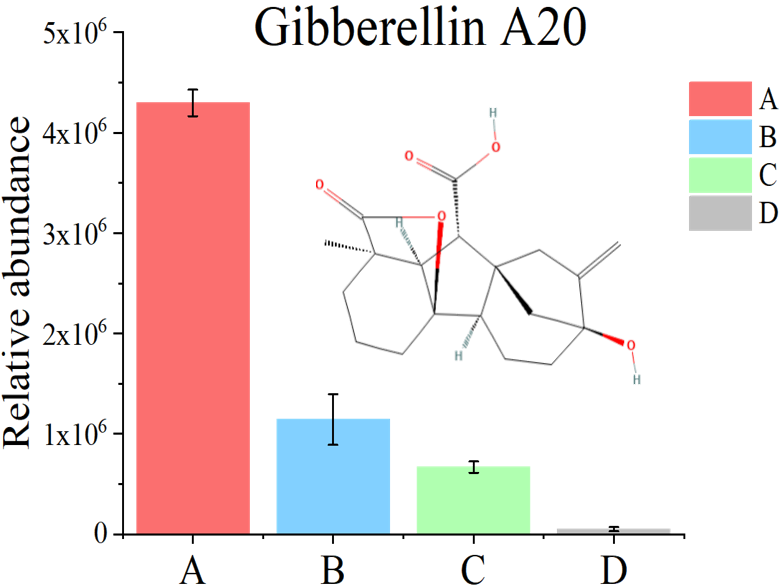 | Plant hormone, regulates stem elongation and seed germination | Down | Down | Down | Diterpenoid | (Liu et al., 2011) |
| 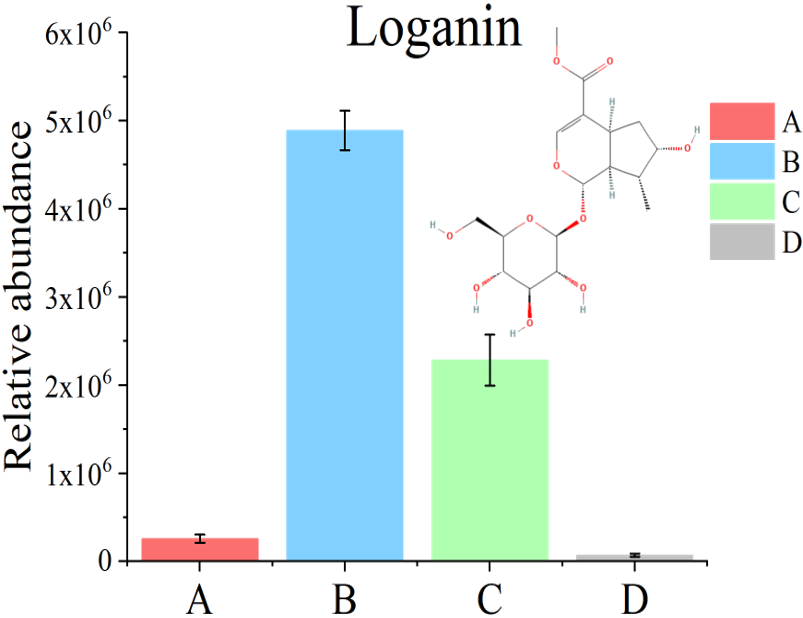 | Anti-inflammatory | Up | Up | Down | Iridoid | (Liu et al., 2020) |

**References**

Antika, L. D., Tasfiyati, A. N., Hikmat, H. and Septama, A. W. (2022). Scopoletin: a review of its source, biosynthesis, methods of extraction, and pharmacological activities, *Z Naturforsch C J Biosci*. 77, 303-316. doi: [10.1515/znc-2021-0193](https://doi.org/10.1515/znc-2021-0193).

Cavalca, L. B., Atlason, U. A., Trofin, A., Ribeiro, C. M., Pavan, F. R., Deuss, P. J., et al. (2024). Selectivity and Activity of Benzene-1,2,4-triol and its Dimers as Antimicrobial Compounds Against Xanthomonas citri subsp. citri, *Chempluschem*. 89: e202300616. doi: [10.1002/cplu.202300616](https://doi.org/10.1002/cplu.202300616).

Chauhan, M., Barot, R., Yadav, R., Joshi, K., Mirza, S., Chikhale, R., et al. (2024). The Mycobacterium tuberculosis Cell Wall: An Alluring Drug Target for Developing Newer Anti-TB Drugs-A Perspective, *Chem Biol Drug Des*. 104: e14612. doi: [10.1111/cbdd.14612](https://doi.org/10.1111/cbdd.14612).

Gao, X., Li, X., Zhang, C. and Bai, C. (2024). Scopoletin: a review of its pharmacology, pharmacokinetics, and toxicity, *Front Pharmacol*. 15: 1268464. doi: [10.3389/fphar.2024.1268464](https://doi.org/10.3389/fphar.2024.1268464).

Kang, C. W., Han, Y. E., Kim, J., Oh, J. H., Cho, Y. H. and Lee, E. J. (2017). 4-Hydroxybenzaldehyde accelerates acute wound healing through activation of focal adhesion signalling in keratinocytes, *Sci Rep*. 7: 14192. doi: [10.1038/s41598-017-14368-y](https://doi.org/10.1038/s41598-017-14368-y).

Kang, M., Choi, W., Yoo, S. H., Nam, S., Shin, P., Kim, K. K., et al (2021). Modulation of Inflammatory Pathways and Adipogenesis by the Action of Gentisic Acid in RAW 264.7 and 3T3-L1 Cell Lines, *J Microbiol Biotechnol*. 31, 1079-1087. doi: [10.4014/jmb.2105.05004](https://doi.org/10.4014/jmb.2105.05004).

Khalil, A., Bramucci, A. R., Focardi, A., Le Reun, N., Willams, N. L. R., Kuzhiumparambil, U., et al. (2024). Widespread production of plant growth-promoting hormones among marine bacteria and their impacts on the growth of a marine diatom, *Microbiome*. 12: 205. doi: [10.1186/s40168-024-01899-6](https://doi.org/10.1186/s40168-024-01899-6).

Kiba, T., Mizutani, K., Nakahara, A., Takebayashi, Y., Kojima, M., Hobo, T., et al. (2023). The trans-zeatin-type side-chain modification of cytokinins controls rice growth, *Plant Physiol*. 192, 2457-2474. doi: [10.1093/plphys/kiad197](https://doi.org/10.1093/plphys/kiad197).

Kimani, B. G., Kerekes, E. B., Szebenyi, C., Krisch, J., Vagvolgyi, C., Papp, T., et al. (2021). In Vitro Activity of Selected Phenolic Compounds against Planktonic and Biofilm Cells of Food-Contaminating Yeasts, *Foods*. 10: 1652. doi: [10.3390/foods10071652](https://doi.org/10.3390/foods10071652).

Kratky, M., Konecna, K., Janousek, J., Brablikova, M., Jandourek, O., Trejtnar, F., et al. (2019). 4-Aminobenzoic Acid Derivatives: Converting Folate Precursor to Antimicrobial and Cytotoxic Agents, *Biomolecules*. 10: 9. doi: [10.3390/biom10010009](https://doi.org/10.3390/biom10010009).

Lee, J., Choi, J., Han, H. Y., Kim, W. S., Song, H., Byun, E., et al. (2020). 4-Hydroxybenzaldehyde Restricts the Intracellular Growth of Toxoplasma gondii by Inducing SIRT1-Mediated Autophagy in Macrophages, *Korean J Parasitol*. 58, 7-14. doi: [10.3347/kjp.2020.58.1.7](https://doi.org/10.3347/kjp.2020.58.1.7).

Liu, S., Shen, H., Li, J., Gong, Y., Bao, H., Zhang, J., et al. (2020). Loganin inhibits macrophage M1 polarization and modulates sirt1/NF-kappaB signaling pathway to attenuate ulcerative colitis, *Bioengineered*. 11: 628-639. doi: [10.1080/21655979.2020.1774992](https://doi.org/10.1080/21655979.2020.1774992).

Liu, Y., Xu, Y., Xiao, J., Ma, Q., Li, D., Xue, Z., et al. (2011). OsDOG, a gibberellin-induced A20/AN1 zinc-finger protein, negatively regulates gibberellin-mediated cell elongation in rice, *J Plant Physiol*. 168: 1098-105. doi: [10.1016/j.jplph.2010.12.013](https://doi.org/10.1016/j.jplph.2010.12.013).

Montagner, C., De Souza, S. M., Groposoa, C., Delle Monache, F., Smania, E. F. A. and Smania, A. J. (2008). Antifungal activity of coumarins, *Z Naturforsch C J Biosci*. 63: 21-8. doi: [10.1515/znc-2008-1-205](https://doi.org/10.1515/znc-2008-1-205).

Pejon, L. S., Oliveira, V. D. C., Amorim, A. A., Raffaini, J. C., Arruda, C. N. F. D. and Pires-De-Souza, F. D. C. P. (2023). Antimicrobial effect of phytosphingosine in acrylic resin, *Braz Dent J*. 34, 107-114. doi: [10.1590/0103-6440202305357](https://doi.org/10.1590/0103-6440202305357).

Robbins, L., Balaram, A., Dejneka, S., Mcmahon, M., Najibi, Z., Pawlowicz, P., et al. (2023). Heterologous production of the D-cycloserine intermediate O-acetyl-L-serine in a human type II pulmonary cell model, *Sci Rep*. 13: 8551. doi: [10.1038/s41598-023-35632-4](https://doi.org/10.1038/s41598-023-35632-4).

Skroza, D., Simat, V., Vrdoljak, L., Jolic, N., Skelin, A., Cagalj, M., et al. (2022). Investigation of Antioxidant Synergisms and Antagonisms among Phenolic Acids in the Model Matrices Using FRAP and ORAC Methods, *Antioxidants (Basel)*. 11: 1784. doi: [10.3390/antiox11091784](https://doi.org/10.3390/antiox11091784).

Sun, Y., Ren, G., Shi, Q., Zhu, H., Zhou, N., Kong, X., et al. (2023). Identification of a Novel Coumarins Biosynthetic Pathway in the Endophytic Fungus Fusarium oxysporum GU-7 with Antioxidant Activity, *Appl Environ Microbiol*. 89: e0160122. doi: [10.1128/aem.01601-22](https://doi.org/10.1128/aem.01601-22).

Sung, M., Lim, S., Park, S., Choi, Y. and Kim, S. (2024). Anti-inflammatory effects of phytosphingosine-regulated cytokines and NF-kB and MAPK mechanism, *Cell Mol Biol (Noisy-le-grand)*. 70, 22-30. doi: [10.14715/cmb/2024.70.9.3](https://doi.org/10.14715/cmb/2024.70.9.3).

Zhang, B., Tian, M., Qiu, Y., Wu, J., Cui, C., Liu, S., et al. (2025). Glucuronolactone Restores the Intestinal Barrier and Redox Balance Partly Through the Nrf2/Akt/FOXO1 Pathway to Alleviate Weaning Stress-Induced Intestinal Dysfunction in Piglets, *Antioxidants (Basel)*. 14: 352. doi: [10.3390/antiox14030352](https://doi.org/10.3390/antiox14030352).

Zhang, J., Chai, X., Zhao, F., Hou, G. and Meng, Q. (2022). Food Applications and Potential Health Benefits of Hawthorn, *Foods*. 11: 2861. doi: [10.3390/foods11182861](https://doi.org/10.3390/foods11182861).

Zhu, L., Sun, S., Wu, W., Zhang, Y., Lin, C. and Ji, L. (2023). Xanthotoxol alleviates secondary brain injury after intracerebral hemorrhage by inhibiting microglia-mediated neuroinflammation and oxidative stress, *Neurochirurgie*. 69: 101426. doi: [10.1016/j.neuchi.2023.101426](https://doi.org/10.1016/j.neuchi.2023.101426).
