# Supplementary material for: Metabolic response of Brevibacterium epidermidis TRM83610 to NaCl stress
Source: Front Microbiol. 2026 Feb 6;17:1754185. doi: 10.3389/fmicb.2026.1754185 (PMC12920567; doi:10.3389/fmicb.2026.1754185)
Supplement: Supplementary file 1 [file Supplementary_file_1.zip › Supplementary material/Table_S2_Functional DMs.pdf]

Table S2 Significantly enriched functional DMs

	Biological function	Regulation (Group A as control)			Class	References
		B	C	D		
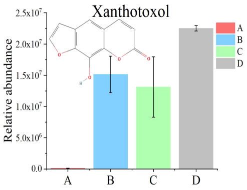	Antioxidan	Up	Up	Up	Coumarin derivative	(Zhu et al., 2023)
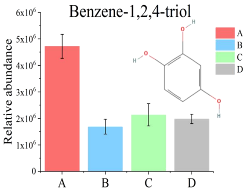	Antioxidant Antibacterial	Down	Down	Down	Phenol derivative	(Cavalca et al., 2024)
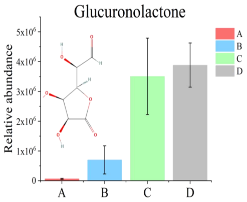	Antioxidant	Up	Up	Up	Lactone	(Zhang et al., 2025)
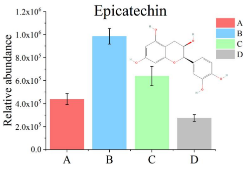	Yeast inhibitor, Antioxidant	Up	Up	Down	Flavonoid	(Kimani et al., 2021, Zhang et al., 2022)
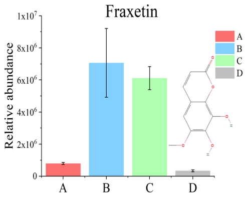	Antioxidant, Anti-inflammat ory, Antibacterial	Up	Up	Down	Coumarin derivative	(Sun et al., 2023, Montagner et al., 2008)
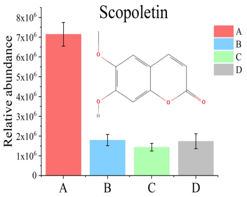	Antibacterial, Antioxidant	Down	Down	Down	Coumarin derivative	(Antika et al., 2022, Gao et al., 2024, Skroza et al., 2022)

Continued table S2 Significantly enriched functional DMs

	Biological function	Regulation (Group A as control)			Class	References
		B	C	D		
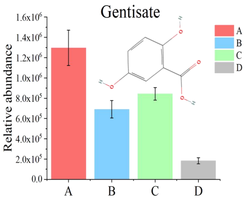	Antioxidant, Anti-inflammatory	Down	Down	Down	Phenolic acid	(Skroza et al., 2022, Kang et al., 2021)
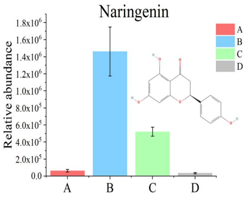	Antioxidant, Anti-inflammatory	Up	Up	Nodiff	Flavonoid	(Kang et al., 2021)
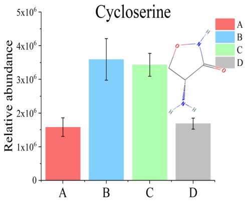	Antibiotic, Inhibits bacterial cell wall synthesis	Up	Up	Nodiff	Cyclic amino acid	(Chauhan et al., 2024, Robbins et al., 2023)
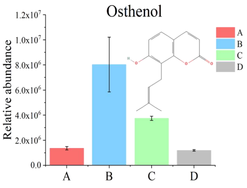	Anti-inflammatory, Antibacterial or Anticancer	Up	Up	Nodiff	Coumarin derivative	(Montagner et al., 2008)
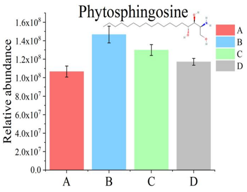	Antibacterial, Anti-inflammatory	Up	Up	Nodiff	Sphingolipid	(Pejon et al., 2023, Sung et al., 2024)
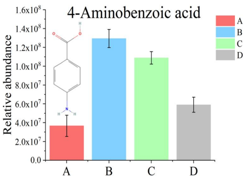	Antibacterial	Up	Up	Nodiff	Aminobenzoic acid	(Kratky et al., 2019)

Continued table 2 Significantly enriched functional DMs

	Biological function	Regulation (Group A as control)			Class	References
		B	C	D		
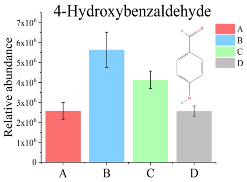	Antibacterial	Up	Up	Nodiff	Phenolic aldehyde	(Kang et al., 2017, Lee et al., 2020)
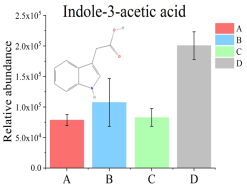	Plant growth hormone (Auxin), regulates cell elongation and differentiation	Up	Up	Up	Indole derivative	(Khalil et al., 2024)
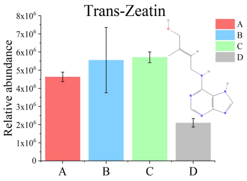	Plant hormone, promotes cell division	Up	Up	Down	Adenine derivative	(Kiba et al., 2023)
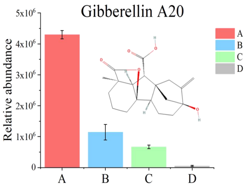	Plant hormone, regulates stem elongation and seed germination	Down	Down	Down	Diterpenoid	(Liu et al., 2011)
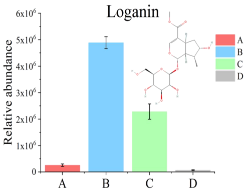	Anti-inflammatory	Up	Up	Down	Iridoid	(Liu et al., 2020)

References

Antika, L. D., Tasfiyati, A. N., Hikmat, H. and Septama, A. W. (2022). Scopoletin: a review of its source, biosynthesis, methods of extraction, and pharmacological activities, *Z Naturforsch C J Biosci*. 77, 303-316. doi: 10.1515/znc-2021-0193.

Cavalca, L. B., Atlason, U. A., Trofin, A., Ribeiro, C. M., Pavan, F. R., Deuss, P. J., et al. (2024). Selectivity and Activity of Benzene-1,2,4-triol and its Dimers as Antimicrobial Compounds Against Xanthomonas citri subsp. citri, *Chempluschem*. 89: e202300616. doi: 10.1002/cplu.202300616.

Chauhan, M., Barot, R., Yadav, R., Joshi, K., Mirza, S., Chikhale, R., et al. (2024). The Mycobacterium tuberculosis Cell Wall: An Alluring Drug Target for Developing Newer Anti-TB Drugs-A Perspective, *Chem Biol Drug Des*. 104: e14612. doi: 10.1111/cbdd.14612.

Gao, X., Li, X., Zhang, C. and Bai, C. (2024). Scopoletin: a review of its pharmacology, pharmacokinetics, and toxicity, *Front Pharmacol*. 15: 1268464. doi: 10.3389/fphar.2024.1268464.

Kang, C. W., Han, Y. E., Kim, J., Oh, J. H., Cho, Y. H. and Lee, E. J. (2017). 4-Hydroxybenzaldehyde accelerates acute wound healing through activation of focal adhesion signalling in keratinocytes, *Sci Rep*. 7: 14192. doi: 10.1038/s41598-017-14368-y.

Kang, M., Choi, W., Yoo, S. H., Nam, S., Shin, P., Kim, K. K., et al (2021). Modulation of Inflammatory Pathways and Adipogenesis by the Action of Gentisic Acid in RAW 264.7 and 3T3-L1 Cell Lines, *J Microbiol Biotechnol*. 31, 1079-1087. doi: 10.4014/jmb.2105.05004.

Khalil, A., Bramucci, A. R., Focardi, A., Le Reun, N., Willams, N. L. R., Kuzhiumparambil, U., et al. (2024). Widespread production of plant growth-promoting hormones among marine bacteria and their impacts on the growth of a marine diatom, *Microbiome*. 12: 205. doi: 10.1186/s40168-024-01899-6.

Kiba, T., Mizutani, K., Nakahara, A., Takebayashi, Y., Kojima, M., Hobo, T., et al. (2023). The trans-zeatin-type side-chain modification of cytokinins controls rice growth, *Plant Physiol*. 192, 2457-2474. doi: 10.1093/plphys/kiad197.

Kimani, B. G., Kerekes, E. B., Szebenyi, C., Krisch, J., Vagvolgyi, C., Papp, T., et al. (2021). In Vitro Activity of Selected Phenolic Compounds against Planktonic and Biofilm Cells of Food-Contaminating Yeasts, *Foods*. 10: 1652. doi: 10.3390/foods10071652.

Kratky, M., Konecna, K., Janousek, J., Brablikova, M., Jandourek, O., Trejtnar, F., et al. (2019). 4-Aminobenzoic Acid Derivatives: Converting Folate Precursor to Antimicrobial and Cytotoxic Agents, *Biomolecules*. 10: 9. doi: 10.3390/biom10010009.

Lee, J., Choi, J., Han, H. Y., Kim, W. S., Song, H., Byun, E., et al. (2020). 4-Hydroxybenzaldehyde Restricts the Intracellular Growth of Toxoplasma gondii by Inducing SIRT1-Mediated Autophagy in Macrophages, *Korean J Parasitol*. 58, 7-14. doi: 10.3347/kjp.2020.58.1.7.

Liu, S., Shen, H., Li, J., Gong, Y., Bao, H., Zhang, J., et al. (2020). Loganin inhibits macrophage M1 polarization and modulates sirt1/NF-kappaB signaling pathway to attenuate ulcerative colitis,

Bioengineered. 11: 628-639. doi: 10.1080/21655979.2020.1774992.

Liu, Y., Xu, Y., Xiao, J., Ma, Q., Li, D., Xue, Z., et al. (2011). OsDOG, a gibberellin-induced A20/AN1 zinc-finger protein, negatively regulates gibberellin-mediated cell elongation in rice, *J Plant Physiol*. 168: 1098-105. doi: 10.1016/j.jplph.2010.12.013.

Montagner, C., De Souza, S. M., Groposoa, C., Delle Monache, F., Smania, E. F. A. and Smania, A. J. (2008). Antifungal activity of coumarins, *Z Naturforsch C J Biosci*. 63: 21-8. doi: 10.1515/znc-2008-1-205.

Pejon, L. S., Oliveira, V. D. C., Amorim, A. A., Raffaini, J. C., Arruda, C. N. F. D. and Pires-De-Souza, F. D. C. P. (2023). Antimicrobial effect of phytosphingosine in acrylic resin, *Braz Dent J*. 34, 107-114. doi: 10.1590/0103-6440202305357.

Robbins, L., Balaram, A., Dejneka, S., Mcmahon, M., Najibi, Z., Pawlowicz, P., et al. (2023). Heterologous production of the D-cycloserine intermediate O-acetyl-L-serine in a human type II pulmonary cell model, *Sci Rep*. 13: 8551. doi: 10.1038/s41598-023-35632-4.

Skroza, D., Simat, V., Vrdoljak, L., Jolic, N., Skelin, A., Cagalj, M., et al. (2022). Investigation of Antioxidant Synergisms and Antagonisms among Phenolic Acids in the Model Matrices Using FRAP and ORAC Methods, *Antioxidants (Basel)*. 11: 1784. doi: 10.3390/antiox11091784.

Sun, Y., Ren, G., Shi, Q., Zhu, H., Zhou, N., Kong, X., et al. (2023). Identification of a Novel Coumarins Biosynthetic Pathway in the Endophytic Fungus Fusarium oxysporum GU-7 with Antioxidant Activity, *Appl Environ Microbiol*. 89: e0160122. doi: 10.1128/aem.01601-22.

Sung, M., Lim, S., Park, S., Choi, Y. and Kim, S. (2024). Anti-inflammatory effects of phytosphingosine-regulated cytokines and NF-kB and MAPK mechanism, *Cell Mol Biol (Noisy-le-grand)*. 70, 22-30. doi: 10.14715/cmb/2024.70.9.3.

Zhang, B., Tian, M., Qiu, Y., Wu, J., Cui, C., Liu, S., et al. (2025). Glucuronolactone Restores the Intestinal Barrier and Redox Balance Partly Through the Nrf2/Akt/FOXO1 Pathway to Alleviate Weaning Stress-Induced Intestinal Dysfunction in Piglets, *Antioxidants (Basel)*. 14: 352. doi: 10.3390/antiox14030352.

Zhang, J., Chai, X., Zhao, F., Hou, G. and Meng, Q. (2022). Food Applications and Potential Health Benefits of Hawthorn, *Foods*. 11: 2861. doi: 10.3390/foods11182861.

Zhu, L., Sun, S., Wu, W., Zhang, Y., Lin, C. and Ji, L. (2023). Xanthotoxol alleviates secondary brain injury after intracerebral hemorrhage by inhibiting microglia-mediated neuroinflammation and oxidative stress, *Neurochirurgie*. 69: 101426. doi: 10.1016/j.neuchi.2023.101426.
